# The clinical, genomic, and microbiological profile of invasive multi-drug resistant *Escherichia coli* in a major teaching hospital in the United Kingdom

**DOI:** 10.1099/mgen.0.001122

**Published:** 2023-10-30

**Authors:** William L. Hamilton, Suny Coscione, Mailis Maes, Ben Warne, Lindsay J. Pike, Fahad A. Khokhar, Beth Blane, Nicholas M. Brown, Theodore Gouliouris, Gordon Dougan, M. Estée Török, Stephen Baker

**Affiliations:** ^1^​ University of Cambridge, Department of Medicine, Cambridge Biomedical Campus, Hills Road, UK; ^2^​ Cambridge University Hospitals NHS Foundation Trust, Cambridge Biomedical Campus, Hills Road, Cambridge CB2 0QQ, UK; ^3^​ Wellcome Sanger Institute, Wellcome Trust Genome Campus, Hinxton, CB10 1RQ, UK; ^4^​ Cambridge Institute for Therapeutic Immunology and Infectious Disease, Jeffrey Cheah Biomedical Centre, Puddicombe Way, Cambridge CB2 0AW, UK; ^5^​ University of Cambridge, Department of Veterinary Medicine, Madingley Road, Cambridge, CB3 0ES, UK; ^6^​ Clinical Microbiology and Public Health Laboratory, Cambridge Biomedical Campus, Hills Road, Cambridge CB2 0QQ, UK

**Keywords:** Antimicrobial resistance, whole genome sequencing, genomic epidemiology, *E. coli*, monoclonal antibody, Gram negative, multidrug resistant, clinical microbiology, healthcare-associated infections

## Abstract

*
Escherichia coli* is a ubiquitous component of the human gut microbiome, but is also a common pathogen, causing around 40, 000 bloodstream infections (BSI) in the United Kingdom (UK) annually. The number of *E. coli* BSI has increased over the last decade in the UK, and emerging antimicrobial resistance (AMR) profiles threaten treatment options. Here, we combined clinical, epidemiological, and whole genome sequencing data with high content imaging to characterise over 300 *E. coli* isolates associated with BSI in a large teaching hospital in the East of England. Overall, only a limited number of sequence types (ST) were responsible for the majority of organisms causing invasive disease. The most abundant (20 % of all isolates) was ST131, of which around 90 % comprised the pandemic O25b:H4 group. ST131-O25b:H4 isolates were frequently multi-drug resistant (MDR), with a high prevalence of extended spectrum β-lactamases (ESBL) and fluoroquinolone resistance. There was no association between AMR phenotypes and the source of *E. coli* bacteraemia or whether the infection was healthcare-associated. Several clusters of ST131 were genetically similar, potentially suggesting a shared transmission network. However, there was no clear epidemiological associations between these cases, and they included organisms from both healthcare-associated and non-healthcare-associated origins. The majority of ST131 isolates exhibited strong binding with an anti-O25b antibody, raising the possibility of developing rapid diagnostics targeting this pathogen. In summary, our data suggest that a restricted set of MDR *E. coli* populations can be maintained and spread across both community and healthcare settings in this location, contributing disproportionately to invasive disease and AMR.

## Data Summary

The ENA accession codes for the 322 isolates analysed in this study are included in Table S7.

**Impact Statement**

We found that the pandemic ST131-O25b:H4 lineage was a common cause of *E. coli* bloodstream infections in the East of England, and frequently carried multiple antimicrobial resistance mechanisms including extended spectrum β-lactamase production and fluoroquinolone resistance. The conserved antigenicity of ST131-O25b:H4 raises the possibility of developing targeted immune therapeutics and rapid diagnostics for this common and frequently multi-drug resistant pathogen.

## Introduction

*Escherichia coli* (* E. coli *) is a ubiquitous commensal of the human gut that can also cause a spectrum of invasive disease [1]. Globally, out of an estimated 13.7 million infection-related deaths in 2019, *E. coli* caused the second highest number of deaths among 33 investigated bacterial pathogens, at 950,000 deaths (after *Staphylococcus aureus* with 1.1 million deaths) [2]. Bloodstream infections (BSI) caused by *E. coli* cause significant morbidity and mortality [2, 3], and the incidence is increasing year on year (Fig. S1, available in the online version of this article) [4]. *E. coli* has become the commonest cause of BSI in the United Kingdom (UK), causing around 40,000 BSI in the UK annually [5]. BSI can trigger acute systemic inflammatory response syndromes that can cause severe end-organ damage and death. Therefore, the prompt recognition, diagnosis and initiation of effective treatment, including antimicrobials, is essential. Gram-negative bacteraemia is becoming increasingly recognised as a public health threat and interventions aimed at reducing its incidence, including infections caused by *E. coli *, are a strategic priority in the UK [6].

Antimicrobial resistance (AMR) limits the therapeutic options for many infections, including BSI caused by Gram-negative bacteria such as *E. coli* [7]. As *E. coli* occupy the gut they are a common sink and source for AMR genes, which can catalyse resistance to a wide array of antimicrobials [8–10]. There is evidence from a Danish cohort that *E. coli* plays a key role in driving the infant gut microbiome towards higher abundance of AMR-associated genes [11]. Understanding the production and proliferation of AMR among microbial communities is a key challenge in clinical microbiology. The relationships between *E. coli* genomic diversity, including the mobile genetic elements that can transport AMR genes, and their clinical and epidemiological characteristics, merit further investigation. These analyses require integration of genomics with clinical microbiology, which can be challenging in healthcare settings.

*E. coli* sequence type (ST)−131, serotype O25b:H4, has spread internationally and is responsible for a significant burden of extended spectrum β-lactamase (ESBL) *E. coli* disease in the UK and globally, in both healthcare and community settings [12–19], in the food chain [20] and in animals [21]. This pandemic lineage possesses virulence mechanisms [22] and is associated with *bla*-CTX-M β-lactamase production and fluoroquinolone resistance. Three ST131 clades have been described (A, B, and C), which can be further divided into subclades [23]. Since the 2000s, clade C became the globally dominant lineage; clade C2 is associated with *bla*-CTX-M-15, and clade C1-M27 with *bla*-CTX-M-27 [24]. There is frequent movement of β-lactamase genes between plasmids and the chromosome, and even closely related ST131 isolates can harbour substantial variation in their complement of plasmid-mediated AMR genes [25]. The genetic architecture underlying AMR in *E. coli* ST131, and how this propagates between individuals and relates to both asymptomatic carriage and invasive disease, is incompletely understood.

Antibacterial immune therapeutics and rapid diagnostic testing are promising tools against invasive bacterial disease, particularly in the context of spreading and intensifying AMR, and a shortage of novel antibacterial agents. Anti-O25b vaccines and diagnostics have been proposed to tackle ST131 [26–28]; however, their design and implementation relies on understanding the genetic variation present in bacterial target genes and populations. This emphasises the importance of linking genomics with clinical, microbiological and epidemiological data.

Here, we aimed to determine the clinical, genomic, and microbiological profile of invasive multi-drug resistant (MDR) *E. coli* in the UK, by studying patients with *E. coli* bloodstream infections in a major hospital in the East of England. All BSI *E. coli* isolates were whole genome sequenced and underwent a detailed phylogenetic analysis to determine the genomic epidemiology of these infections in hospital and community settings. Lastly, exploiting a commercial monoclonal antibody (mAb) against the O25b antigen, we aimed to understand antigen conservation in ST131 isolates to add to data supporting the development of immune therapeutics and diagnostics for common AMR/MDR organisms associated with BSI.

## Methods

### Study setting and processing of clinical specimens

All blood culture isolates were collected from patients at Cambridge University Hospitals NHS Foundation Trust (CUH) in the East of England. Comprising Addenbrooke’s and the Rosie Hospitals, CUH has approximately 1,100 acute beds and provides secondary care services for Cambridge and the surrounding area, serving a population of approximately 580 000 people. It also provides tertiary care services (including infectious diseases, oncology, haematology, solid organ transplantation, neurosurgery, paediatrics and neonatology) for the East of England.

Blood culture sets (BacT/ALERT, bioMérieux) were obtained from patients as clinically indicated. All samples were processed in the UK Health Security Agency (UKHSA) Clinical Microbiology and Public Health Laboratory (CMPHL), which operates from the CUH site. All routine microbiological techniques were performed according to UK Standards for Microbiology Investigations (UK SMIs) [29]. Species identification was determined using mass spectrometry (MALDI-TOF MS, Bruker Daltonics). Antimicrobial susceptibility testing was determined using the disc diffusion method, with breakpoints determined by the British Society for Antimicrobial Chemotherapy (BSAC) criteria in use at the time (this study pre-dates the laboratory change to the European Committee on Antimicrobial Susceptibility Testing (EUCAST) methodology). All blood culture isolates were prospectively stored in glycerol broth at −80 °C.

*E. coli* isolates of interest were identified from a database of all culture results from CUH, exported from the CMPHL laboratory information system, and filtered based on sample type and species. This provided data on sample collection and result dates, patient identifiers and antimicrobial susceptibility results determined by the clinical laboratory. All available *E. coli* isolates from samples collected between 1 December 2016 and 31 December 2017 were included in the study.

### Genome sequencing

Whole-genome sequencing was performed on all available stored isolates, except for duplicate isolates defined as blood culture samples from the same patient occurring within 90 days of the first isolate collection date. Standard DNA extraction methods were used as described in Supplementary Methods. DNA libraries were prepared using the WGS Ultra II library kit and sequenced on an Illumina HiSeq platform (Illumina) using a standard protocol at the Wellcome Sanger Institute.

### Bioinformatics

#### QC filters

The following quality control (QC) filtering was applied to genome sequences: total genome length <6 MB, contig count <150, Kraken organism 1 is *E. coli *, and percentage reads mapping to Kraken organism 1>30 % (Figs S3-S5). The resulting phylogenetic tree included a long branch with four outlier samples, which were removed for subsequent analyses to yield the final analysis set (Fig. S6).

#### Core genome alignment and producing phylogenetic tree

Core genome alignment, tree building, single nucleotide polymorphism (SNP) distance matrix, AMR gene detection, plasmid detection, multilocus sequence typing and *in silico* OH serotyping were performed on the Wellcome Sanger Institute high performance computing (HPC) network. Downstream analyses and visualisations were produced using the R programming language with *tidyverse* packages. Further details can be found in Supplementary Methods. Briefly, core genome alignment was performed using *roary* [30], requiring genes to be shared across 99 % of isolates (Table S1). A maximum likelihood phylogenetic tree was generated from the core genome alignment using IQTREE [31], using a GTR+G substitution model [32] with ultrafast bootstrapping for branch support [33]. The resulting phylogenetic tree was initially visualised and manipulated using Microreact [34] and final figures presented in the paper were produced using *ggtree* [35]. A SNP distance matrix was produced using *snp-dists* [36].

#### AMR gene and plasmid detection

AMR genes and variants were detected from the Comprehensive Antibiotic Resistance Database (CARD) database, downloaded from: https://card.mcmaster.ca/, version 3.1.1 [37]. Antibiotic Resistance Identification By Assembly (ARIBA) [38] was used to prepare the CARD database and analyse the samples. *plasmidfinder* was used to identify plasmids [39], also prepared using ARIBA.

#### Multilocus sequence typing (MLST) and OH serotyping

MLST typing and OH serotyping were performed using *srst2* [40].

#### Clinical and epidemiological data linkage

Prior to pseudonymisation for sequencing, the patient Medical Record Number was used to link records to the CUH electronic health record system (Epic, Epic Systems) and to locally held infection control databases created for submission to the Public Health England (PHE, now UK Health Security Agency) mandatory surveillance programme for hospital acquired infections.

Demographic data including gender, age, main specialty, etc. were extracted from Epic, whilst suspected infectious focus was obtained from the infection control database. Patient identifiable data were stored securely on the CUH server. Only pseudonymised, non-identifiable data were transferred to the Wellcome Sanger Institute. Categorisation of infection (healthcare-onset, healthcare-associated or community-onset) was made by accessing individual patient records and making an assessment based on adapted criteria, described in Supplementary Methods. To assess epidemiological association between selected cases, bed movements during hospital admission were reviewed. Strong epidemiological linkage was defined as two patients having been on the same ward and bay at any stage during admission. Weak epidemiological linkage was defined as having been on the same ward or under the same clinical specialty up to a week apart, without close contact based on bed location.

#### Antibody binding and image analysis

The monoclonal antibody used for high-content antibody binding assay was KM467, an IgG1 antibody based on VH and VL sequences of 3E9-11 [41]. The synthesis of this antibody and methodology used for the high content imaging assay have been previously described [42]. The full protocol for antibody binding is described in Supplementary Methods. Briefly, bacterial colonies were picked, cultured overnight in LB broth, diluted, washed and incubated with the KM467 antibody in CellCarrier-96 Ultra plates (PerkinElmer), followed by addition of Alexa Fluor 647 labelled anti-human IgG and DAPI. Plates were imaged on an Opera Phenix confocal microscope (PerkinElmer) using the Alexa Fluor 647 and DAPI channels with the 63× water immersion objective. Sixteen fields and three Z-stacks were imaged per well. The image analysis was carried out using PerkinElmer Harmony (v4.9). The final result output was categorical, based on whether there was antibody binding or no binding imaged on the plate.

#### Statistical analysis

Logistic regression to test for associations between variables and 30 day mortality were performed in R using a generalized linear model. The R code took the general form:

model <- glm(mortality_30_days ~.,family=binomial(link='logit'), data=metadata

Summary(model)

anova(model, test=‘Chisq’)

Where the ‘metadata’ file comprised columns of metadata such as age, sex, etc.

Clusters of ST131 were defined from the SNP difference matrix produced from the *snp-dists* package, using *scipy.cluster.hierarchy* functions *dendrogram*, *linkage*, and *fcluster* to identify clusters within a specified genetic distance from each-other, corresponding to the intended SNP difference.

The two-sided Wilcoxon rank sum test for non-parametric statistical hypothesis testing, and Fisher’s exact test, were implemented in R. Two by two contingency tables were used to calculate the sensitivity, specificity, positive predictive value, and negative predictive value of ST131 and O25 antigen status for ESBL production and ciprofloxacin susceptibility (Supplementary Methods).

## Results

### Study population and clinical outcome

**Fig. 1.**
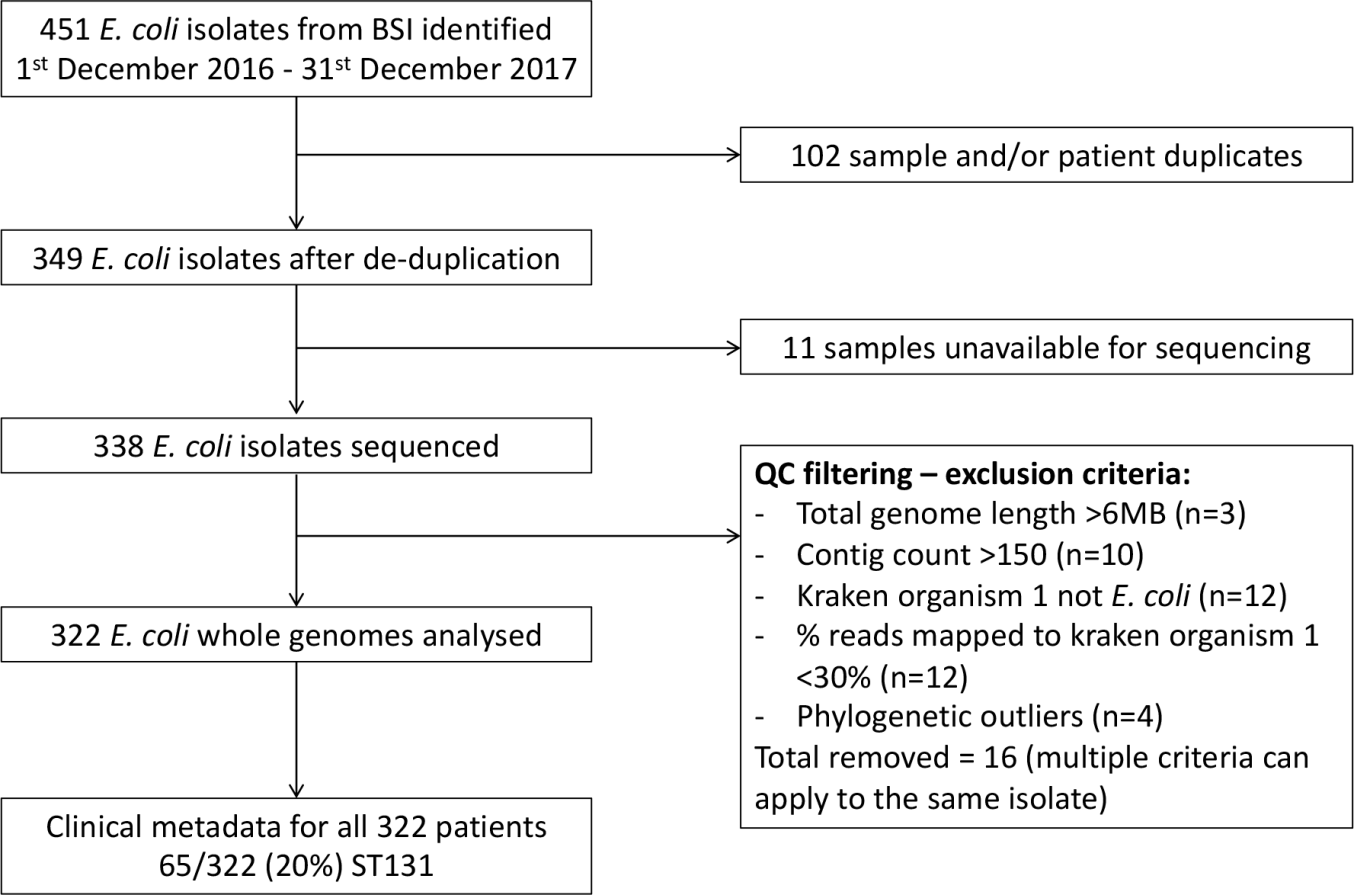
Study flow diagram. This shows the number of *E. coli* samples included in the study and in the final analysis. A total of 451 *E. coli* isolates from 349 individuals were identified within the study period (1 December 2016 – 31 December 2017), of which 338 were sequenced and 322 passed sequencing quality control (QC) filtering and were used for the final analysis. From this, 65/322 (20 %) samples were identified as being sequence type 131, which underwent anti-O25b antibody testing.

A total of 451 *E. coli* BSI isolates at Cambridge University Hospitals NHS Foundation Trust were identified within the designated study dates (1 December 2016 to 31 December 2017). After sample de-duplication, there were 349 patients with *E. coli* BSI represented by at least one sample, and whole genome sequencing was performed on at least one *E. coli* isolate originating from 338 patients (Fig. 1). After genome quality control (QC) filtering, the final analysis group comprised 322 *E. coli* BSI isolates with high-quality whole genome sequence information and associated metadata.

The epidemiological characteristics of the study population are shown in Table 1. Median patient age was 74 years old (interquartile range [IQR], 58–85 years), with 174/322 (54 %) female. Where patient address information was available (*n*=161/322, 50 %), the majority of patients (118/161, 73 %) lived within the local county (Cambridgeshire) (Fig. S2). The most commonly identified source of the bacteraemia (151/322; 47 %) was a primary urinary tract infection (UTI). Overall, 128/322 (40 %) of cases were categorised as being ‘community-onset, healthcare-associated’ (CO-HA); 105/322 (33 %) were ‘community-onset, community-associated’ (CO-CA), and 83/322 (26 %) were ‘hospital-onset, healthcare-associated’ (HO-HA). Median patient age was similar for both categories of community-onset infections (CO-HA: 75 years, CO-CA: 76 years), and slightly younger for hospital-onset, healthcare-associated infections (67 years; *P*=0.002 for difference between community- vs hospital-onset infection age distributions, two-sided Wilcoxon rank sum test).

**Table 1.** Study population epidemiological characteristics. Only patients associated with high quality *E. coli* genomes included in the study analysis (*n*=322) are presented. IQR=Interquartile Range

		Study population (*n*=322)
**Age (years**)	Median (IQR) – all Median (IQR) – adults	74 (58–85) 75 (60–85)
**Number of adults, paediatric and neonatal patients**	Adults Paediatric Neonatal	313 (97 %) 4 (1.2 %) 5 (16 %)
**Sex**	Female Male	174 (54 %) 148 (46 %)
**Bacteraemia source**	Urinary tract Hepatobiliary Gastrointestinal (other than hepatobiliary) Other Unknown	151 (47 %) 57 (18 %) 18 (5.6 %) 52 (16 %) 44 (14 %)
**Infection classification**	Community-onset, healthcare-associated Community-onset, non-healthcare-associated Hospital-onset, healthcare-associated Unclassified	128 (40 %) 105 (33 %) 83 (26 %) 6 (1.9 %)
**30 day mortality**	Total Adult	40/322 (12 %) 38/313 (12 %)

In total, 40/322 (12 %) patients died within 30 days of the onset of bacteraemia. The association between 30 day mortality in adult patients with age, sex, healthcare association, bacteraemia source, whether the *E. coli* had an ESBL or AmpC phenotype, and whether the organism was ST131, was investigated by logistic regression. Only age and bacteraemia source were significantly associated with 30 day mortality (*P*=0.049 and *P*=1.6×10^−5^, respectively). Overall, 18/111 (16 %) adults aged >80 years old died within 30 days of BSI onset, compared with 20/202 (9.9 %) in patients aged <80 years old (Table S2). BSI with a urinary tract source was strongly associated with reduced odds of 30 day mortality: 5/149 (3.4 %) adults with urinary tract source died within 30 days compared with 33/164 (20 %) adults with any other primary source of infection.

### Phenotypic antimicrobial susceptibility profile

**Fig. 2.**
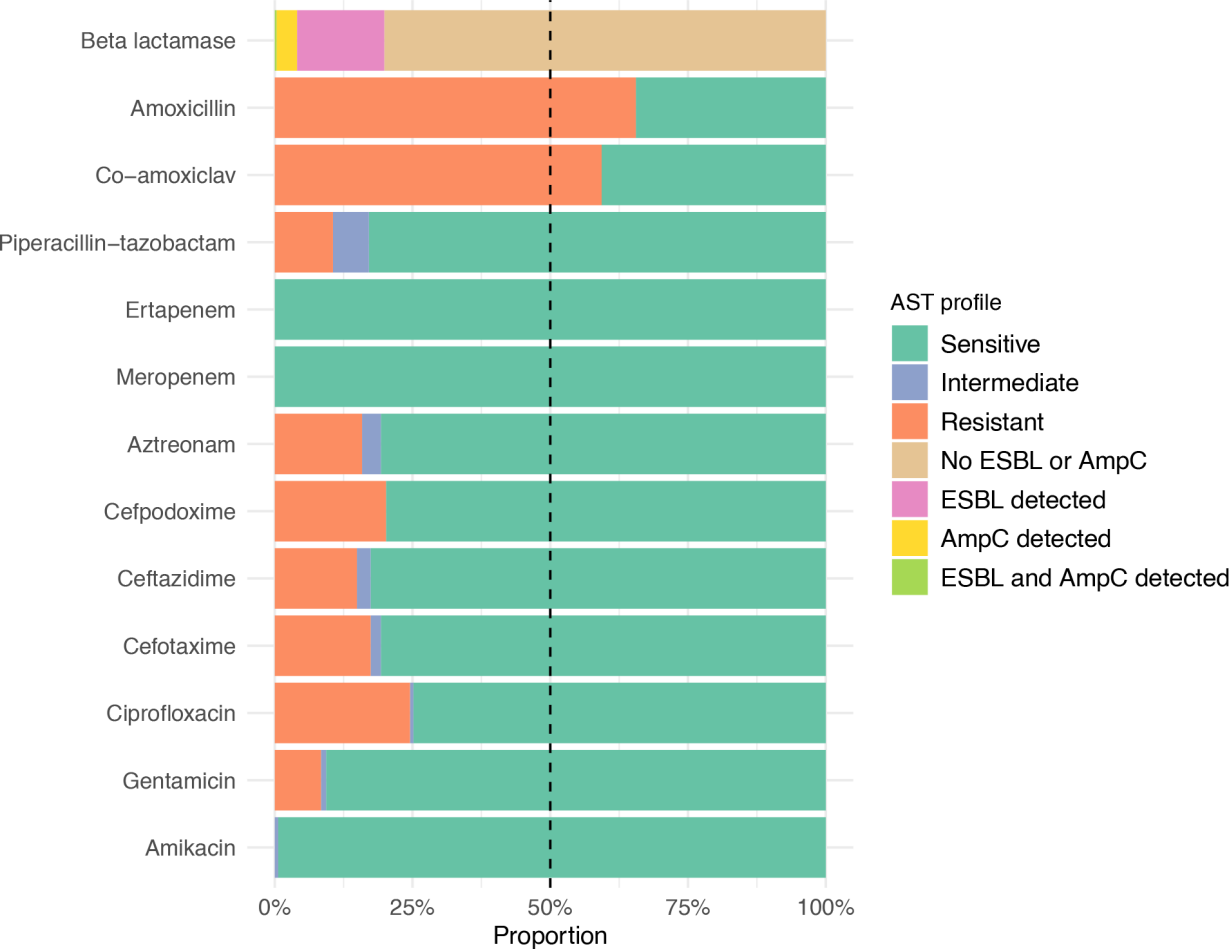
Phenotypic antimicrobial susceptibility profile for all 322 isolates. Antimicrobial susceptibility was determined by disc diffusion with break points interpreted using BSAC zone-size cut-offs. Isolates that were resistant to cefpodoxime were selected for further testing to determine whether an ESBL or AmpC phenotype was present.

The phenotypic antimicrobial susceptibility test (AST) profile for the 322 *E. coli* isolates, measured by disc diffusion, is shown in Fig. 2. There was a high prevalence of resistance to the β-lactam + β-lactamase inhibitor combination amoxicillin + clavulanic acid (co-amoxiclav), with 191 (59 %) isolates being resistant. Resistance to piperacillin + tazobactam, another β-lactam + β-lactamase inhibitor combination, was lower (34 [11 %], 21 [6.5 %] and 267 [83 %] isolates were resistant, intermediate and susceptible, respectively). An ESBL and/or AmpC phenotype was detected in 52 (16 %) and 13 (4 %) isolates, respectively (with a single isolate generating both phenotypes); no carbapenem resistant organisms were identified. Resistance to aminoglycosides was low: 27 (8.4 %), three (<1 %) and 292 (91 %) isolates were resistant, intermediate and susceptible to gentamicin, respectively. Gentamicin is currently the recommended first-line treatment for complicated UTI in CUH; nearly all isolates were susceptible to amikacin (two [<1 %] and 320 [99 %] were intermediate and susceptible, respectively).

### Genomic characterisation

**Fig. 3.**
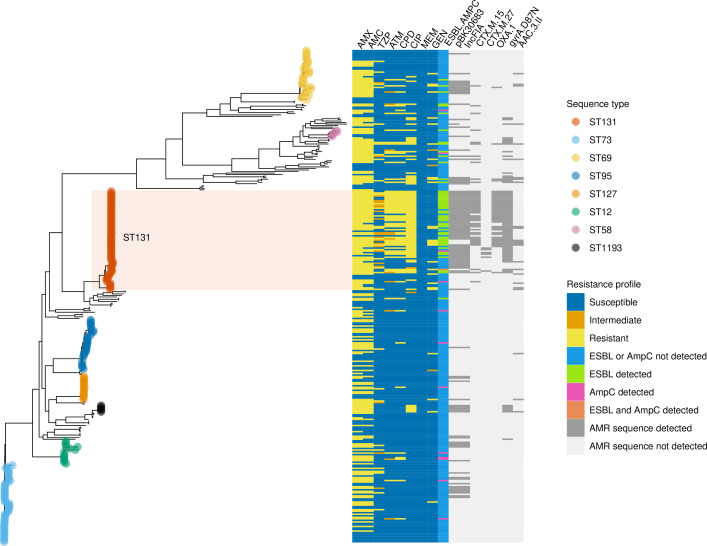
Core genome phylogeny of the *E. coli* study population. Branch tips are coloured by sequence type, showing the eight sequence types with at least five samples. Coloured bars depict phenotypic antimicrobial susceptibility profile (left) and the presence or absence of selected AMR-associated genes and plasmids (right). Sequence type 131 (ST131) is highlighted, which was the most frequent sequence type identified (65/322 [20 %] isolates) and associated with high rates of AMR (Fig. 4). AMR=antimicrobial resistance, AMX=Amoxicillin, AMC=Co-amoxiclav (amoxicillin-clavulanic acid), TZP=piperacillin-tazobactam, ATM=Aztreonam, CPD=Cefpodoxime, CIP=Ciprofloxacin, MEM=Meropenem, GEN=Gentamicin, ESBL=extended spectrum β-lactamase, AMPC=AmpC β-lactamase.

The phylogeny of the 322 *E. coli* isolates, generated by the core genome sequences, is shown in Fig. 3. In total, 75 sequence types were identified; however, the majority were identified at low frequency, with 42 being represented only by a single sample (Table S3, Fig. S7). Just four STs accounted for over half (54%) of all cases: ST131 (65, 20 %), ST73 (51, 16 %), ST69 (33, 10 %), and ST95 (28, 8.7 %). The core genome sequences generated a well-defined population structure, which correlated precisely with assigned ST (Figs S8, S9).

**Fig. 4.**
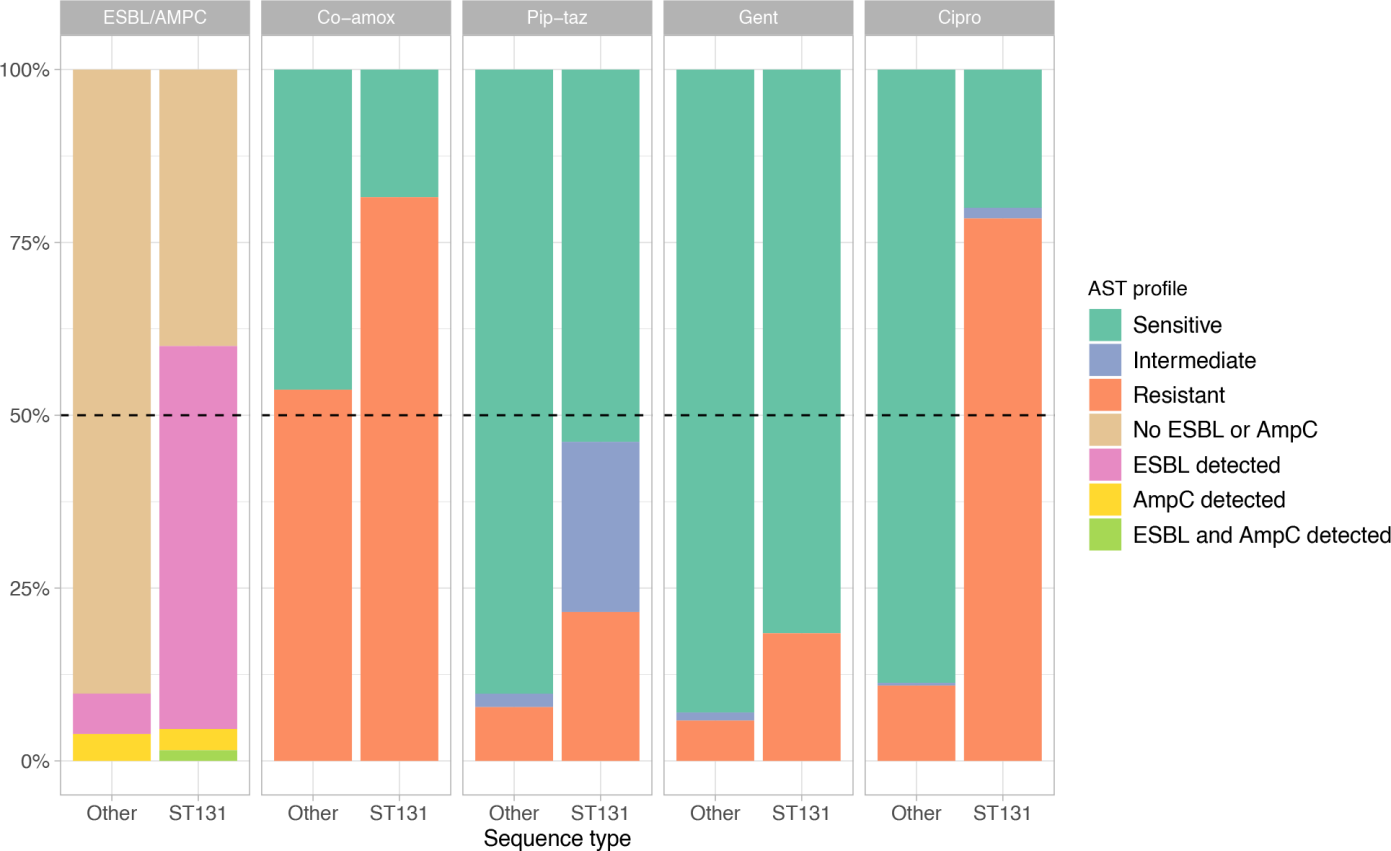
Phenotypic antimicrobial susceptibility profile for selected antimicrobials for ST131 vs non-ST131 isolates. Antimicrobial susceptibility was determined by disc diffusion using BSAC cut-offs for interpretation, and ESBL and AmpC status was determined for cefpodoxime resistant isolates. Isolates are divided into ST131 (*n*=65) and non-ST131 (*n*=257). Results for ESBL or AmpC status (‘ESBL/AMPC’), amoxicillin-clavulanic acid (‘Co-amox’), piperacillin-tazobactam (‘Pip-taz’), gentamicin (‘Gent’) and ciprofloxacin (‘Cipro’) are shown.

ST131 was the most abundant ST and was associated with phenotypic resistance to multiple antimicrobials (Fig. 4). Of the ST131 organisms, 37/65 (57 %) had an ESBL detected, compared with 15/257 (5.8 %) for non-ST131 samples; 51/65 (78 %) were ciprofloxacin resistant, compared with 28/257 (11 %) for non-ST131 isolates. The unadjusted odds ratio (OR) for ST131 being classified as an ESBL producer and ciprofloxacin resistant was 21 (95 % confidence interval, CI, 9.9–47, *P*<10^−5^, Fisher’s exact test) and 29 (95 % CI 14–65, *P*<10^−5^, Fisher’s exact test), respectively. ST131 isolates were more likely to possess AMR resistance-associated genes, variants and plasmids, such as the β-lactamases *bla*-CTX-M-15, *bla*-CTX-M-27, and *bla*-OXA-1, and the plasmid structures pBK30683 and IncFIA, than non-ST131 isolates (Figs 3 and S10). Specifically, 34/65 (52 %) ST131 isolates carried *bla*-CTX-M-15 and 6/65 (9.2 %) carried *bla*-CTX-M-27. In contrast, among non-ST131 isolates (*n*=257), there were 11 (4.3 %) samples with *bla*-CTX-M-15, three (1.2 %) with *bla*-CTX-M-14, and one (0.39 %) with *bla*-CTX-M-1 (Table S4). In total, 71/79 (90 %) of all isolates determined to be phenotypically resistant to ciprofloxacin carried the *gyrA* mutations S83L and D87N and the *parC* mutation S80I, which are among the most common fluoroquinolone resistance-associated mutations in *E. coli* [43, 44]. One out of 241 (0.41 %) isolates that were phenotypically susceptible to ciprofloxacin carried these mutations (Table S5). Among ST131 isolates, 49/51 (96 %) of the phenotypically ciprofloxacin resistant isolates carried the *gyrA*-S83L, *gyrA*-D87N and *parC*-S80I mutations.

### The clinical and epidemiological characteristics of ST131

**Fig. 5.**
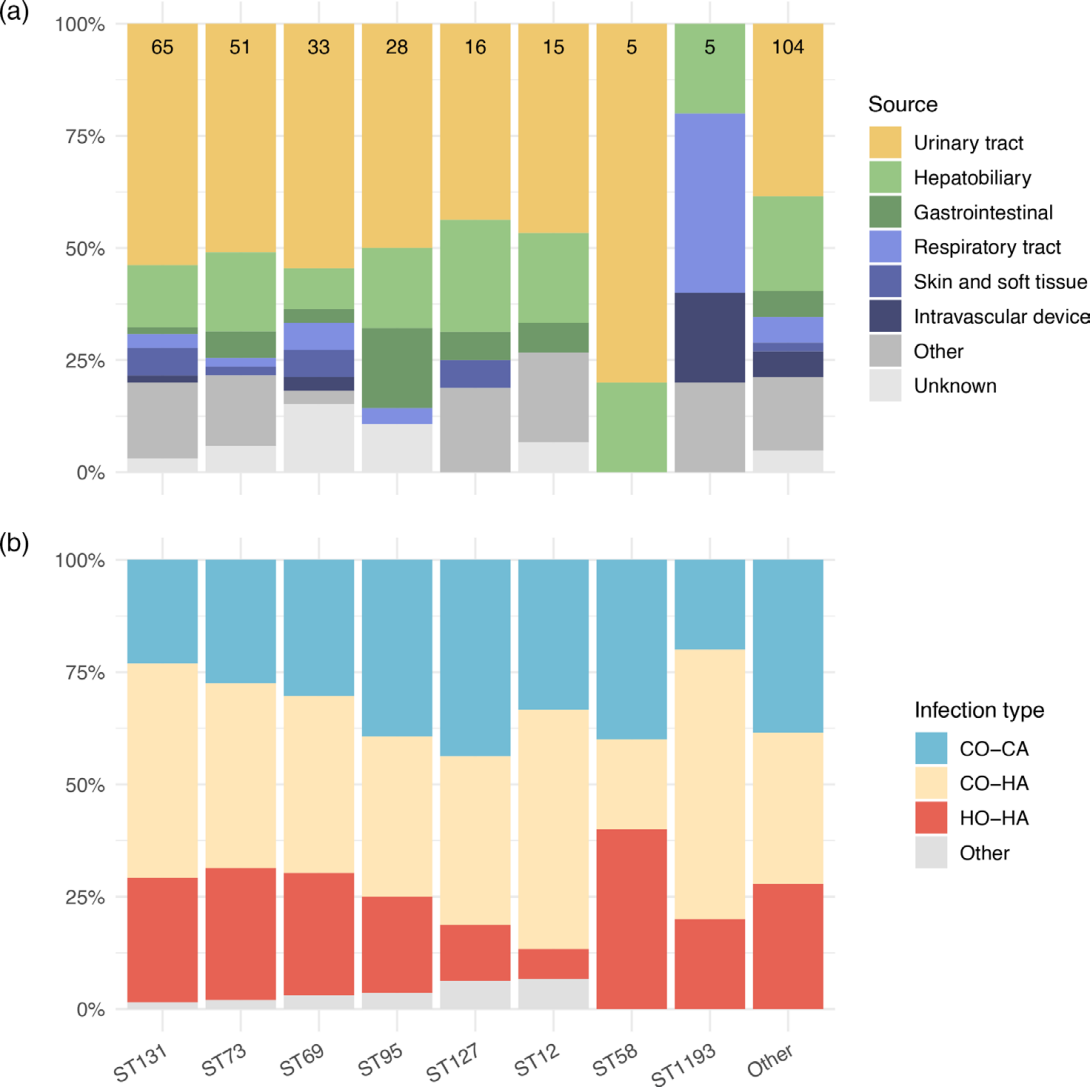
Bacteraemia source and healthcare association class for major sequence types. Showing all sequence types with >5 samples, in size order from highest to lowest left to right, followed by all other isolates. Number of isolates in each sequence type displayed in the top row of bars. CO-CA=community-onset, community-associated; CO-HA=community-onset, healthcare-associated; HO-HA=hospital-onset, healthcare-associated.

There was no association between isolates belonging to ST131 and the likelihood for organisms to be healthcare-associated or the source of bacteraemia (Figs 5 and S11). This observation implies that ST131 can cause invasive disease in both community and healthcare settings. Indeed, all the major STs had a comparable profile of bacteraemia source (most commonly UTI) and the proportion that were healthcare-associated. One exception was ST1193, of which none were associated with UTI; however, this group only contained five isolates.

**Fig. 6.**
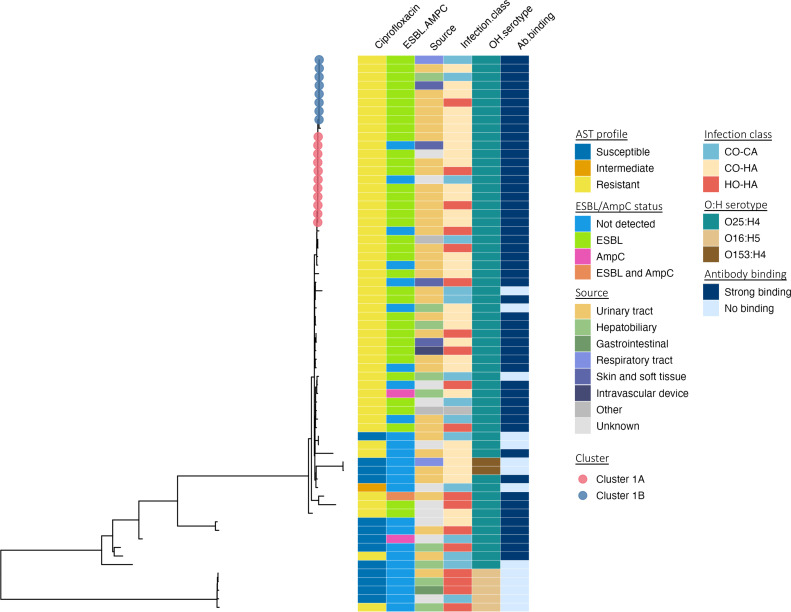
ST131 core genome phylogeny. Sub-tree of the same phylogeny displayed in Fig. 3 showing the 65 ST131 isolates. Branch tips depict the two largest clusters of highly similar ST131 isolates. Coloured bars show, from left to right, ciprofloxacin susceptibility (determined by disc diffusion and BSAC interpretations), ESBL or AmpC status (assayed in cefpodoxime resistant isolates), infection source and classification, *in silico* OH serotype prediction, and the strength of anti-O25b antibody binding. A large monophyletic group of isolates were nearly all ciprofloxacin resistant, mostly possessed an ESBL, and were nearly all serotype O25b:H4 with strong binding to the anti-O25b antibody. The majority of infections were from a urinary source, and a mixture of community- and healthcare-associated cases were represented. CO-CA=community-onset, community associated; CO-HA=community-onset, healthcare-associated; HO-HA=hospital-onset, healthcare-associated.

Disrupting the transmission of AMR within clinical settings is a key goal of infection prevention and control (IPC). We investigated potential epidemiological links between genetically similar ST131 isolates to better understand possible transmission mechanisms. Clusters of highly-similar ST131 isolates were defined with a maximum permitted pairwise SNP difference from the core genome alignment of 17 SNPs within clusters (previously suggested as an epidemiologically relevant cut-off for transmission investigations [45]). We identified two large clusters, referred to as Cluster 1A (11 isolates, median pairwise SNP differences: 4 SNPs [IQR: 2–7, range: 0–12]) and Cluster 1B (eight isolates, median pairwise SNP differences: 2 SNPs [IQR: 1–6, range: 0–14]). One pair of samples in Cluster 1A and three samples in Cluster 1B had zero SNP differences. Both clusters were entirely comprised of ST131-O25b:H4 isolates; all were phenotypically ciprofloxacin resistant and 17/19 had an ESBL producing phenotype; 18/19 carried *bla*-CTX-M-15 and all carried the *gyrA*-S83L, *gyrA*-D87N and *parC*-S80I mutations (Fig. 6). We also noted a smaller group of MDR organisms, ST1193 (five samples in total), in which three samples formed a cluster (with maximum pairwise difference of four SNPs). These ST1193 isolates were serotype O75:H5, not ESBL producers, and were resistant to ciprofloxacin.

Both ST131 clusters included both community- and healthcare-associated cases. On review of patient electronic medical records, no epidemiological links were identified between cases in ST131 Cluster 1B or the ST1193 cluster. In ST131 Cluster 1A, only weak epidemiological links were identified between two sets of patients. One patient pair overlapped the same ward for a single day but were not placed in the same patient bay. Another pair were placed in the same patient bay but did not temporally overlap. None of the hospital acquired cases showed clinical or epidemiological links to other cases within each cluster. In summary, we did not identify any clear signals of direct patient-to-patient transmission of ST131, even for highly similar isolates.

### O:H serotyping *in silico* and *in vitro*

We evaluated the O:H serotype profile of the isolates *in silico*. Ninety-three O and H serotype combinations were identified (Table S6). As with the STs, the distribution was highly skewed; 58 O:H serotypes were represented by only a single sample, while the top five O:H serotypes included 136/322 (42.2 %) of all samples. The most common O:H serotype was O25:H4 (60 isolates, 18.6%); which has been associated with ESBL-producing ST131 isolates in several countries. We found 58/65 (89.2 %) ST131 were O25:H4 in this collection, of which 39/58 (67 %) had an ESBL and/or AmpC phenotype and 50/58 (86 %) were ciprofloxacin resistant. The remaining ST131 isolates were serotype O16:H5 (*n*=5) and O153:H3 (*n*=2), none of which were ESBL or AmpC producers. The two non-ST131 O25:H4 isolates were both ST95 (neither were ESBL or AmpC producers). The 65 ST131 genomes were aligned against an ST131-specific reference genome and a 347 bp fragment of the *pabB* gene was extracted from the alignment (using primers from [46, 47]). This analysis indicated that all O25 isolates from the collection specifically belonged to O25b. All of the ST131 isolates carrying *bla*-CTX-M-15 (*n*=34), *bla*-CTX-M-27 (*n*=6), and *bla*-OXA-1 (*n*=34) were ST131-O25b:H4. Thirty (52 %) and six (10 %) of ST131-O25b:H4 isolates carried *bla*-CTX-M-15 or *bla*-CTX-M-27, respectively; and 50 (86 %) carried the *gyrA*-S83L, *gyrA*-D87N and *parC*-S80I mutations.

The sensitivity and specificity of an organism being ST131 as a marker of phenotypic ESBL production in this *E. coli* cohort was 71 and 90 %, respectively; and the sensitivity and specificity of ST131 as a marker of phenotypic ciprofloxacin resistance was 65 and 94 %, respectively. The positive predictive value (PPV) and negative predictive value (NPV) of an organism being ST131 as a marker of phenotypic ESBL production was 57 and 94 %, respectively, and for phenotypic ciprofloxacin resistance, of 78 and 89 %, respectively. The values for *in silico* O25 antigen serotype status were similar (Supplementary Methods). A negative rapid serology test for ST131-O25b:H4 in *E. coli* BSI could therefore be an early indicator that an isolate is unlikely to be an ESBL producer or ciprofloxacin resistant; a positive result would suggest the isolate was more likely to be an ESBL producer or ciprofloxacin resistant.

**Fig. 7.**
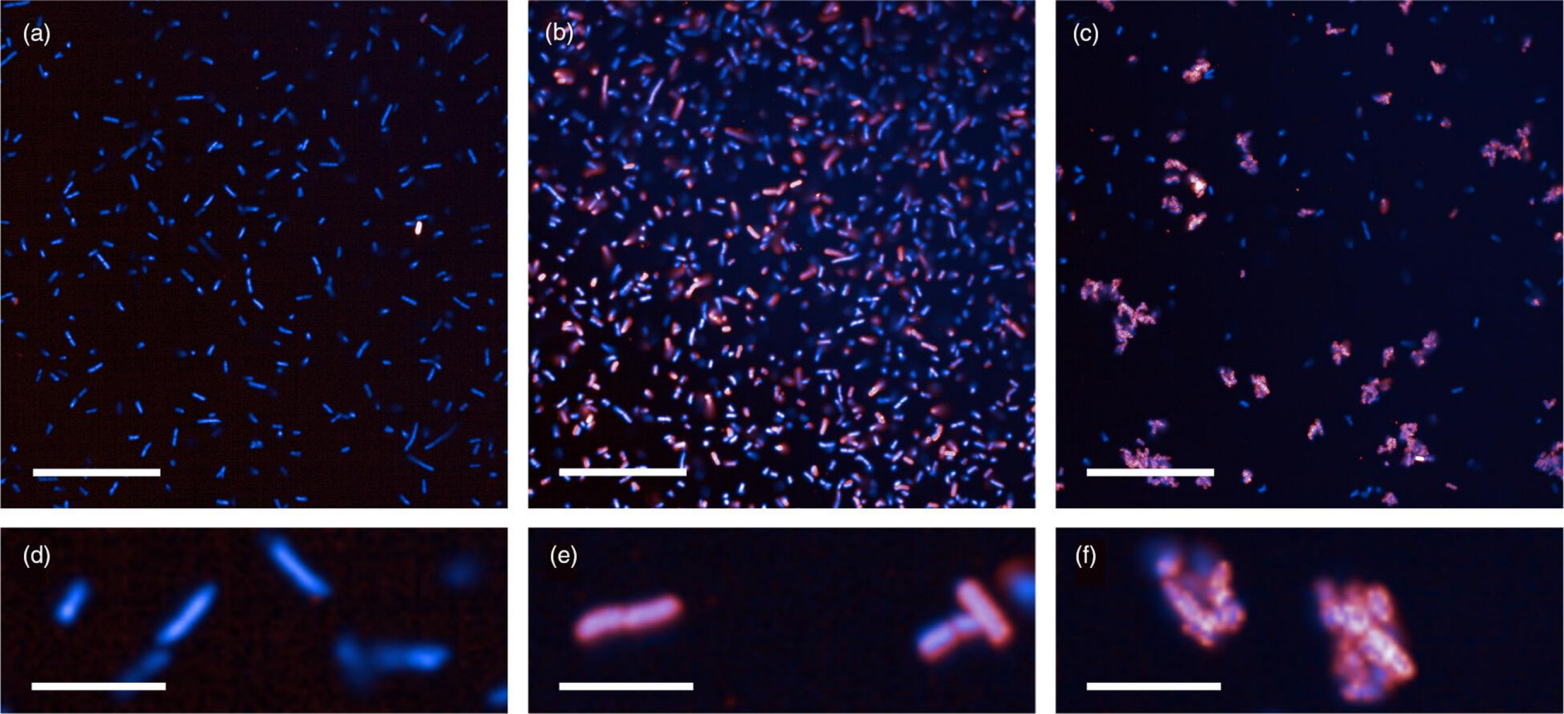
Immunofluorescence of anti-O25b antibody binding for ST131 isolates. Shown in blue (DAPI) nucleoid of *E. coli* ST131; shown in red (Alexa-647) KM-467 anti-O25b antibody. The main phenotypes identified were ‘no binding’ (a, magnified in d), ‘strong binding’ (b, magnified in e), and ‘strong binding with agglutination’ (c, magnified in f). Scale bar: a–c=50 microns, d–f=10 microns.

To further explore the potential for an antibody-based rapid diagnostic test for ST131-O25b:H4, as a potential indicator of ESBL production and fluoroquinolone resistance in *E. coli *, we evaluated the binding strength of an anti-O25b antibody against all ST131 isolates. The majority of isolates had strong (49/65, 75 %) or strong agglutinating (2/65, 3 %) antibody binding (grouped as ‘strong binding’); a minority had no (14/65, 22 %) antibody binding (Fig. 7). All of the strong binding samples were predicted to be serotype O25b:H4 *in silico*. Of the 14 samples with no binding, seven were O25b:H4, five were O16:H5, and two were O153:H4. The clusters of genetically similar, low-diversity *E. coli* ST131 were all O25b:H4 and all exhibited strong antibody binding (Fig. 6).

## Discussion

In this study, we combined multiple data sources including clinical, epidemiological, genomic and antibody binding data to characterise *E. coli* BSI in a large UK hospital. Of the 322 isolates included in the study, 12 % patients died within 30 days of their bacteraemia, with increasing age and non-urinary source associated with higher odds of death. By disc diffusion testing, 59 % isolates were resistant to co-amoxiclav, 16 % had an ESBL, and 4 % had an AmpC. At the time the samples were collected, co-amoxiclav was the first-line recommended treatment for complex UTI in the hospital; this has since been switched to gentamicin, for which resistance was low (around 90 % isolates were susceptible to gentamicin). There were no carbapenem resistant organisms identified.

Consistent with previous work [13], ST131 was the most frequent sequence type, accounting for around 20 % of all isolates. The majority of ST131 (around 90 %) were serotype O25b:H4, which was associated with phenotypic ciprofloxacin resistance, ESBL production, and low-diversity clusters. Despite being highly similar across the core genome, these clusters generally did not have discernible epidemiological links between them, consistent with previous studies [48]. This raises the question of how genetically similar *E. coli* isolates, including MDR organisms such as clones of ST131-O25b:H4, are spreading between individuals. One possibility is that transmission occurs through contaminated environments, such as fomites. Consistent with this view, *E. coli* ST131 has been found to be prevalent in wastewater samples [49–52], and has been detected in the microbiome of neonates and their mothers [53]. The organisms may then become incorporated into the human microbiome, periodically causing pathology when such organisms enter a new niche, such as the urinary tract. Another possibility is that transmission occurs in pre-hospital settings such as care homes [12].

ST131, when compared with non-ST131 isolates, was commonly associated with both ciprofloxacin resistance and ESBL production; this observation may have potential for use as an initial rapid screening tool for AMR in *E. coli* BSI in this population. The negative predictive value of ST131 for ciprofloxacin resistance and ESBL illustrates the potential for genomic surveillance combined with antimicrobial susceptibility data to identify markers of clinically salient phenotypes, such as AMR, for the development of rapid diagnostics. Screening tests such as this rely on understanding the relationship between the screening target (e.g. O25b) and the phenotype of interest (e.g. ESBL production). Importantly, this may change over time due to ongoing evolutionary processes in microbial populations. For example, an O25b screening test for ESBLs would lose its high negative predictive value if applied in a population in which ESBLs were common in non-O25b *E. coli* isolates. Of note, antimicrobial resistance has expanded in ST131 clade A isolates from Norway [8], and in Australia, clade A isolates carrying *bla*-CTX-M-27 and fluoroquinolone resistance-associated mutations in *gyrA* and *parC* have been identified [54] – previously mainly found in ST131 clade C (containing the O25b:H4 pandemic lineage). A mouse model has suggested that *E. coli* ST131 clade A O16:H5 isolates can cause similar high virulence to ST131-O25b:H4 [55]. In our dataset, only 5/322 (1.6 %) isolates were ST131-O16:H5; none of these isolates were phenotypically ESBL producers and 1/5 was phenotypically ciprofloxacin resistant, though the *gyrA*-D87N mutation was not identified. O25b-based assays may therefore be informative in this population, but have limited longevity and/or geographic application, depending on the latest regional *E. coli* genomic epidemiology. Therefore, ongoing surveillance, integrating molecular profiling, clinical epidemiology, and traditional microbiology, is required to ensure that diagnostic tests and targeted therapies remain reliable, and to identify potential new targets as organisms continually evolve.

We acknowledge several limitations to our study. First, it was a single centre study conducted over 1 year. Sampling longitudinally would allow investigation of evolutionary trends in *E. coli* populations, for example, whether ST131 and specific clades and serotypes are increasing in frequency relative to other sequence types over time, and how new lineages emerge and spread. However, our findings are consistent with other studies [13], which also showed a small number of sequence types, notably ST131, cause the majority of invasive *E. coli* disease. Second, we did not sample from the community to contextualise the BSI *E. coli* genomic diversity; for example, within the gut microbiome or in uncomplicated lower UTI managed in the community. Wastewater sampling suggests that the genomic profile of invasive *E. coli* is highly skewed compared with the commensal *E. coli* population present in the human gut [52]. Finally, there were limitations on what clinical metadata could be retrospectively collected. For example, we were unable to reliably identify whether UTI were catheter-associated, particularly if there had been catheter manipulation in the community prior to admission to hospital. This was often not documented clearly in the electronic medical records, and may represent an opportunity for improved documentation for Gram-negative bacteraemia surveillance going forward.

In summary, we present the clinical, epidemiological, genomic and antibody-binding profile of a cohort of over 300 invasive *E. coli* isolates, identifying clusters of MDR ST131-O25b:H4 organisms that are maintained in both healthcare-associated and community settings. Reducing the spread of these MDR clusters should be a key target for combating AMR. Antigenic correlates of virulence and AMR in a population, such as O25b, could be further investigated for potential vaccines, therapeutics and rapid diagnostic tests.

## Supplementary Data

Supplementary material 1
