# Supplementary material for: The clinical, genomic, and microbiological profile of invasive multi-drug resistant Escherichia coli in a major teaching hospital in the United Kingdom
Source: Microb Genom. 2023 Oct 30;9(10):001122. doi: 10.1099/mgen.0.001122 (PMC10634454; doi:10.1099/mgen.0.001122)
Supplement: Supplementary material 1 [file mgen-9-1122-s001.pdf]

The clinical, genomic, and microbiological profile of invasive multi-drug resistant *Escherichia coli* in a major teaching hospital in the United Kingdom

Supplementary materials

Table of Contents

Supplementary methods ***2***

Bacterial DNA extraction **2**

Bioinformatics **2**

Categorisation of *E. coli* infection class **3**

Calculating anti-O25b sensitivity, specificity, positive and negative predictive values for ESBL production and ciprofloxacin susceptibility **4**

ST131 antibody binding assay **5**

References **5**

Supplementary figures ***6***

Supplementary Figure 1 .... **6**

Supplementary Figure 2 .... **6**

Supplementary Figure 3 .... **7**

Supplementary Figure 4 .... **7**

Supplementary Figure 5 .... **8**

Supplementary Figure 6 .... **8**

Supplementary Figure 7 .... **9**

Supplementary Figure 8 .... **10**

Supplementary Figure 9 .... **11**

Supplementary Figure 10 .... **11**

Supplementary Figure 11 .... **12**

Supplementary tables ***13***

Supplementary Table 1 .... **13**

Supplementary Table 2 .... **13**

Supplementary Table 3 .... **13**

Supplementary Table 4 .... **16**

Supplementary Table 5 .... **17**

Supplementary Table 6 .... **18**

Supplementary Table 7 .... **21**

Supplementary methods

Bacterial DNA extraction

All isolates were streaked from frozen stocks onto Iso-Sensitest or Luria Bertani (LB) agar (Oxoid), and incubated overnight at 37°C in a static incubator. Single colonies were picked into a single well of a 96 deep well plate containing Iso-Sensitest broth, before overnight incubation. Bacteria were pelleted and re-suspended in PBS and 20 µL of 100 mg/mL lysozyme solution. DNA extraction was performed using either the MagNAPure small volume nucleic acid extraction kit (Roche) on the FLOW flex platform (Roche), or using the QIAamp 96 DNA QIAcube HT Kit (Qiagen), according to the manufacturer's instructions. Resultant DNA concentration and purity was quantified using a NanoDrop spectrophotometer (Thermo Fisher Scientific) and a Tapestation 4200 using D1000 ScreenTapes (Agilent Technologies).

Bioinformatics

Command line used for *roary* core genome alignment:

```
roary -e -n -cd 99 -p 8 ../*.gff
```

Command line used for producing phylogenetic tree from the core genome alignment using *iqtree*:

```
iqtree -s core_genome_alignment.aln -m GTR+G -bb 1000
```

Command line for producing SNP distance matrix from the *roary* core genome alignment:

```
snp-dists core_genome_alignment.aln > core_genome_snp_dists.csv -c
```

Command lines using *ariba* to prepare the CARD database (Version 3.1.1, downloaded from https://card.mcmaster.ca/):

```
ariba getref card out.card
ariba prepareref -f out.card.fa -m out.card.tsv prepareref.out.card
```

A generic version of the command line used to analyse the samples using the prepared CARD database is shown below, which was looped through all samples:

```
ariba run prepareref.out.card/ LANEID/LANEID_1.fastq.gz LANEID/LANEID_2.fastq.gz
LANEID_out.card
```

The *ariba* output was summarised as below (generic version of command line):

```
ariba summary out.card.summary LANEID1_out.card/report.tsv
LANEIDn_out.card/report.tsv
```

Specific components of individual ARIBA sample reports were also extracted, e.g. *bla*-CTX-M for subtyping, as follows:

```
egrep -r “CTX-M” */report.tsv
```

plasmidfinder was used to identify plasmids. The *plasmidfinder* database was accessed using:

```
ariba getref plasmidfinder out.plasmidfinder
```

The *plasmdfinder* database was prepared using *ariba*:

```
ariba prepareref -f out.plasmidfinder.fa -m out.plasmidfinder.tsv
prepareref.out.plasmidfinder
```

E. coli MLST alleles were retrieved using:

```
getmlst.py --species 'Escherichia coli#1
```

Where the *E. coli* MLST fasta file used was Escherichia_coli#1.fasta. MLST typing was performed using *srst2*, example command below (looped through each sample):

```
srst2 --input_pe strainA_1.fastq.gz strainA_2.fastq.gz --output strainA_test --log --mlst_db
Escherichia_coli#1.fasta --mlst_definitions profiles_csv --mlst_delimiter _
```

And outputs compiled:

```
srst2 --prev_output *__results.txt --output ecoli_mlst_report
```

In silico OH serotyping was also performed using *srst2*. Example command line used:

```
srst2 --input_pe LANEID/LANEID_1.fastq.gz LANEID/LANEID_2.fastq.gz --output
OH_serotypes --log --gene_db [PATH]/EcOH.fasta
```

And the data compiled:

```
srst2 --prev_output *__results.txt --output ecoli_oh_serotype_report
```

The 65 genomes identified as ST131 using the *srst2* method were mapped against the ST131 reference genome, Escherichia_coli_UPEC_ST131_v0.2. Primers for the *pabB* gene (TCCAGCAGGTGCTGGATCGT and GCGAAATTTTTCGCCGTACTGT) were used for *in silico* PCR for O antigen typing, taken from [1] and [2]. The 347bp amplicon locus was retrieved from the whole genome alignment (positions 2143355 – 2143701 from the reference genome) and inspected in *AliView*.

Categorisation of *E. coli* infection class

Classifications were adapted from mandatory reporting surveillance guidance from UKHSA: https://assets.publishing.service.gov.uk/government/uploads/system/uploads/attachment_data/file/859759/HCAIDCS_Gram_negative_Submission_Form_2020.pdf

Health-care onset was defined as blood culture positive more than 48 hours after admission. **Health-care associated** was defined as blood culture positive less than 48 hours after admission and one of more of the following criteria: hospital admission in previous 28 days, chemotherapy or neutropenia (less than 0.5) in previous 28 days, surgery in previous 28 days, antibiotic prescription in community in previous 28 days, urinary catheter insertion or manipulation in 28 days before admission. **Community onset** was defined as blood culture positive less than 48 hours after admission and nil of above risk factors identified from review of clinical note and GP entries in month before admission.

Calculating anti-O25b sensitivity, specificity, positive and negative predictive values for ESBL production and ciprofloxacin susceptibility

The following contingency tables were used to calculate the sensitivity, specificity, positive predictive value (PPV) and negative predictive value (NPV) for samples carrying the O25 antigen in silico and/or being ST131:

O25 antigen status vs ESBL production:

	ESBL positive	ESBL negative
O25 positive	38	24
O25 negative	14	246

Sensitivity: 73%
Specificity: 91%
PPV: 61%
NPV: 95%

O25 antigen status vs ciprofloxacin susceptibility:

	Cipro resistant	Cipro not resistant
O25 positive	50	12
O25 negative	29	231

Sensitivity: 63%
Specificity: 95%
PPV: 81%
NPV: 89%

ST131 status vs ESBL production:

	ESBL positive	ESBL negative
ST131 positive	37	28
ST131 negative	15	242

Sensitivity: 71%
Specificity: 90%
PPV: 57%
NPV: 94%

ST131 status vs ciprofloxacin susceptibility:

	Cipro resistant	Cipro not resistant
ST131 positive	51	14
ST131 negative	28	229

Sensitivity: 65%
Specificity: 94%
PPV: 78%
NPV: 89%

ST131 antibody binding assay

Bacteria were cultured from storage beads onto LB agar plates. Single colonies were picked and cultured in LB broth overnight at 37°C at 200 RPM. Four additional bacterial isolates known from previous work to have different binding patterns were used as controls. Overnight bacterial cultures were diluted 1:200 in LB broth and 50µl of this bacterial suspension was added per well in CellCarrier-96 Ultra plates (Perkin Elmer) and left in a static incubator for 2 hours at 37°C. The supernatant was discarded and any remaining bacteria fixed with 4% paraformaldehyde (PFA) in phosphate buffered saline (PBS) for 15 minutes at room temperature. After washing with PBS, 100µl of the antibody KM467 (diluted to 1µg/ml in PBS + 1% bovine serum albumin) was added for 1 hour at room temperature. After further wash with PBS, 100µl of 2µg/ml Alexa Fluor 647 Goat Anti-Human IgG (ThermoFisher) plus 2µg/ml DAPI (Thermo Fisher) in PBS + 1% bovine serum albumin were added and the plate was incubated for 30 minutes in the dark. After a final wash with PBS, 50µl of PBS was added to each well and the plate was taken for imaging. The antibody binding assay was repeated fivefold.

References

1. **Johnson JR, Clermont O, Johnston B, Clabots C, Tchesnokova V, *et al.*** Rapid and specific detection, molecular epidemiology, and experimental virulence of the O16 subgroup within Escherichia coli sequence type 131. *J Clin Microbiol* 2014;52:1358–1365.
2. **Clermont O, Dhanji H, Upton M, Gibreel T, Fox A, *et al.*** Rapid detection of the O25b-ST131 clone of Escherichia coli encompassing the CTX-M-15-producing strains. *J Antimicrob Chemother* 2009;64:274–277.

Supplementary figures

Supplementary Figure 1

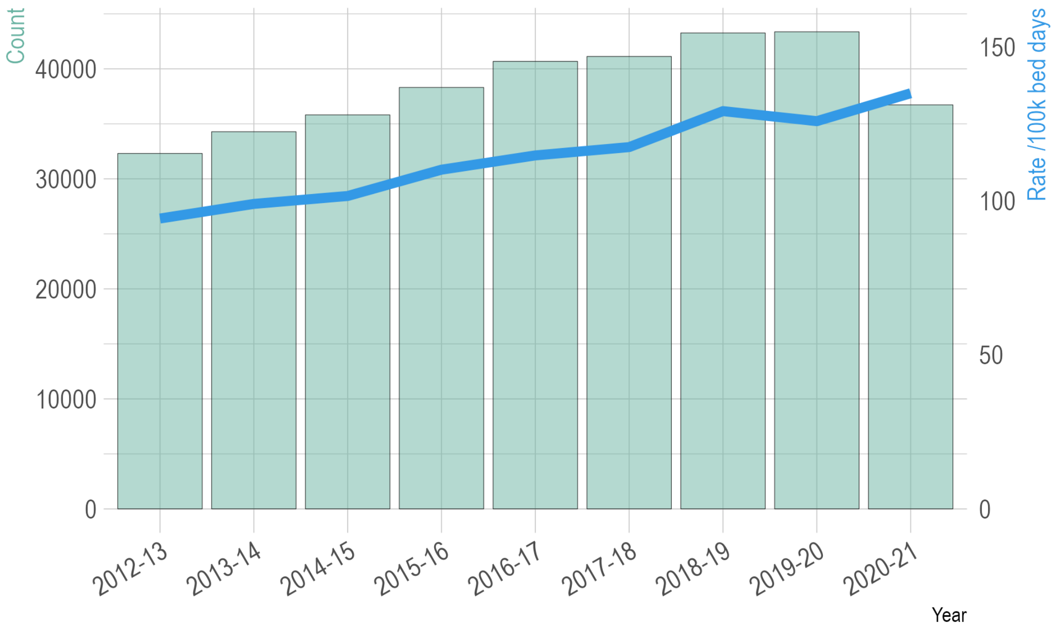

E. coli bacteraemias in NHS acute Trusts 2012-2021. Plotted from data from UK-HSA, 'Escherichia coli bacteraemia: annual data', URL: https://www.gov.uk/government/statistics/escherichia-coli-e-coli-bacteraemia-annual-data, accessed 2023-02-27.

Supplementary Figure 2

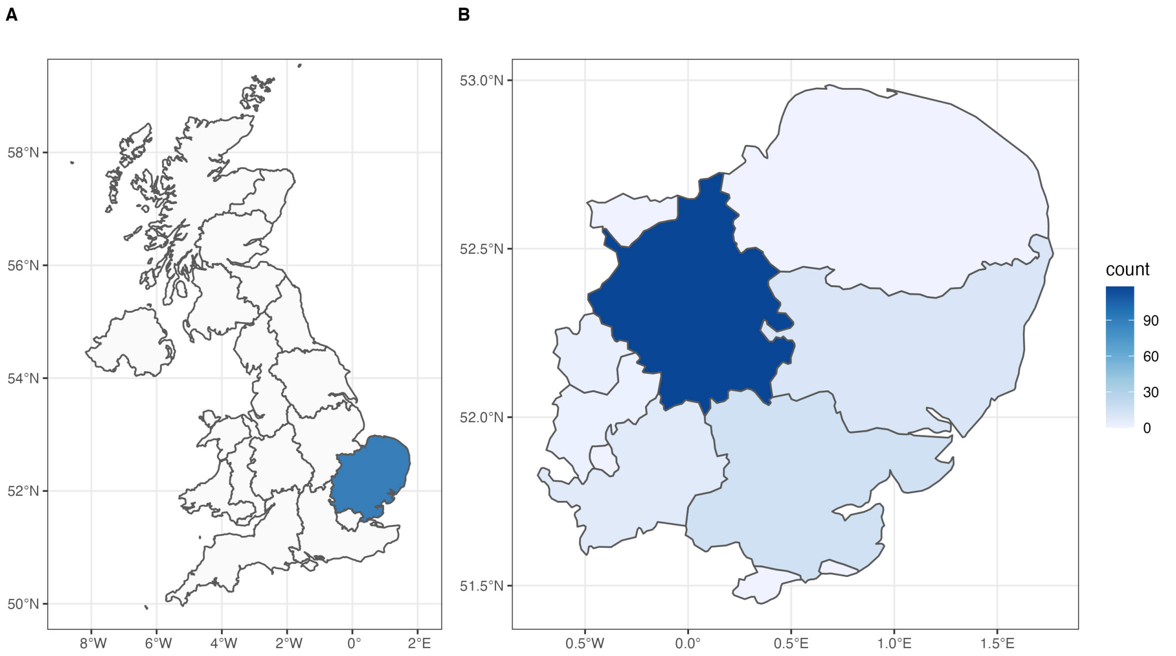

Study location in the United Kingdom (UK) and geographic distribution of E. coli samples. A) Map of the UK with East of England region highlighted in blue. B) Heat map of East of England coloured by sample count used in this study, for the 161 samples in which patient address information was available. 73% of cases were from Cambridgeshire.

Supplementary Figure 3

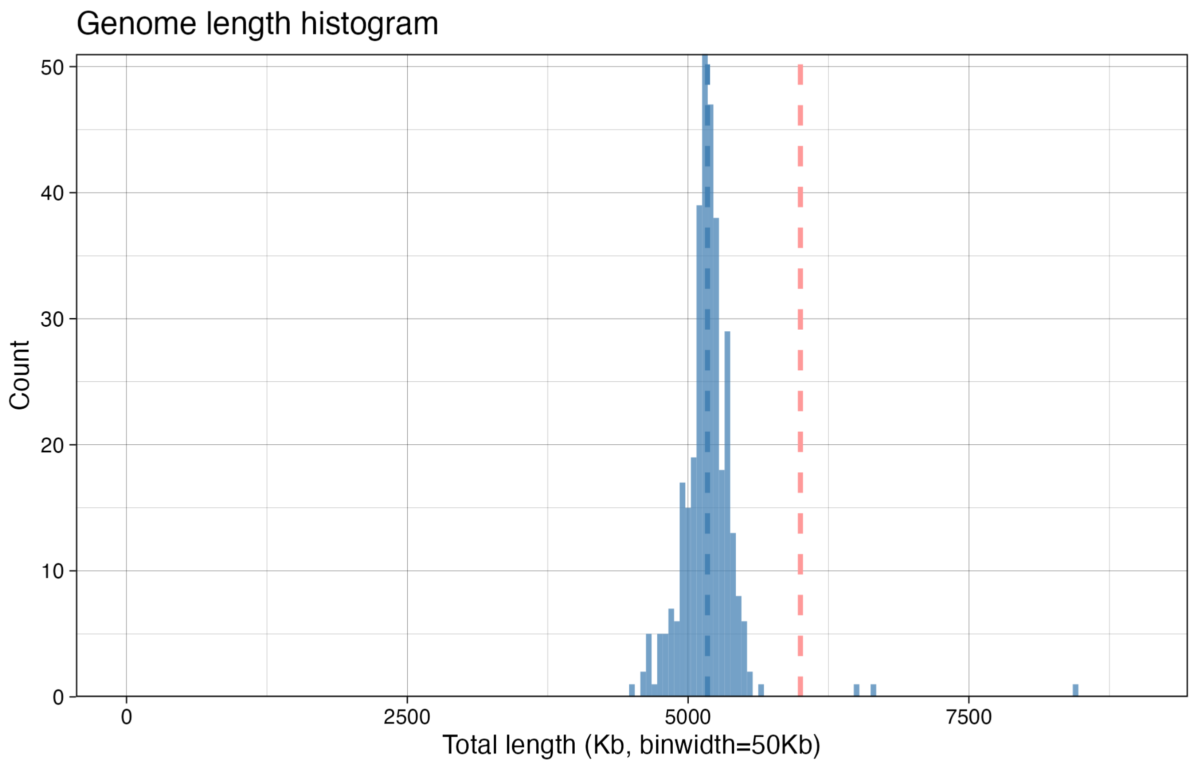

Histogram of bacterial genome length for the 338 samples pre-QC filtering. Dotted vertical lines show median (blue) and QC cut-off (<6MB, red).

Supplementary Figure 4

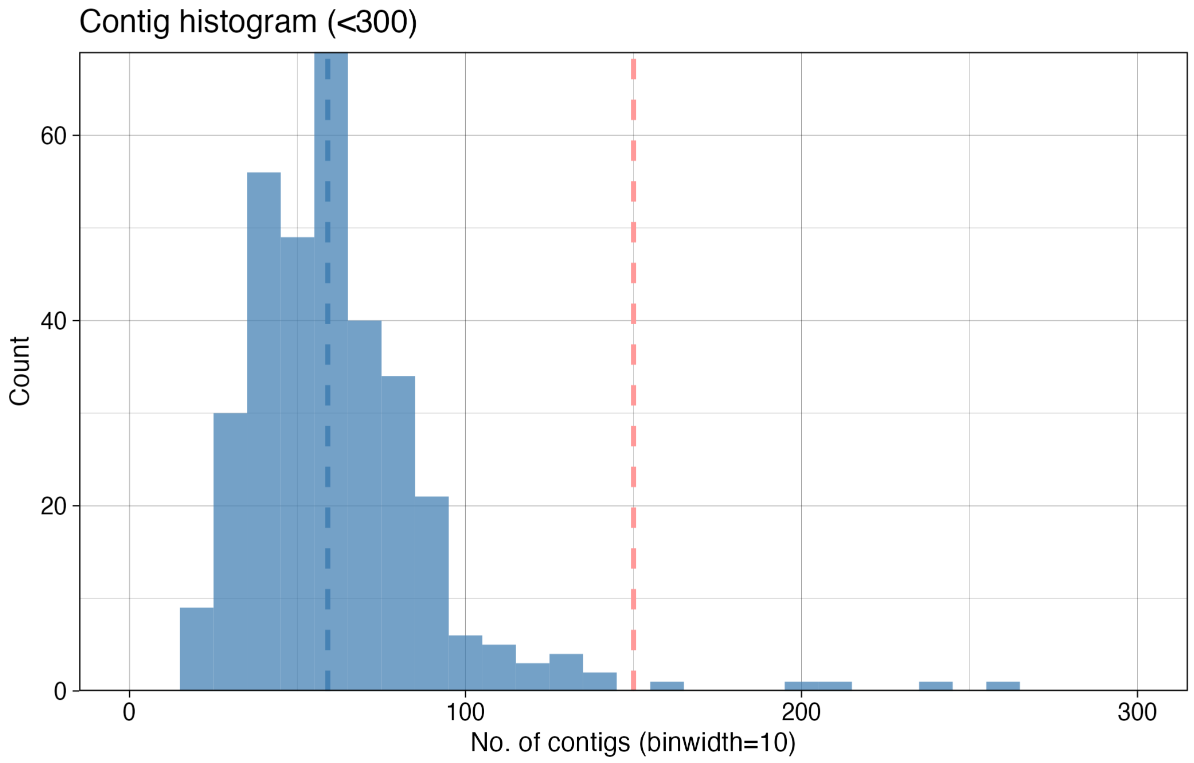

Distribution of number of contigs per sample for the 338 samples pre-QC filtering. 5 outliers with >300 contigs not shown. Dotted vertical lines show median (blue) and QC cut-off (<150 contigs, red).

Supplementary Figure 5

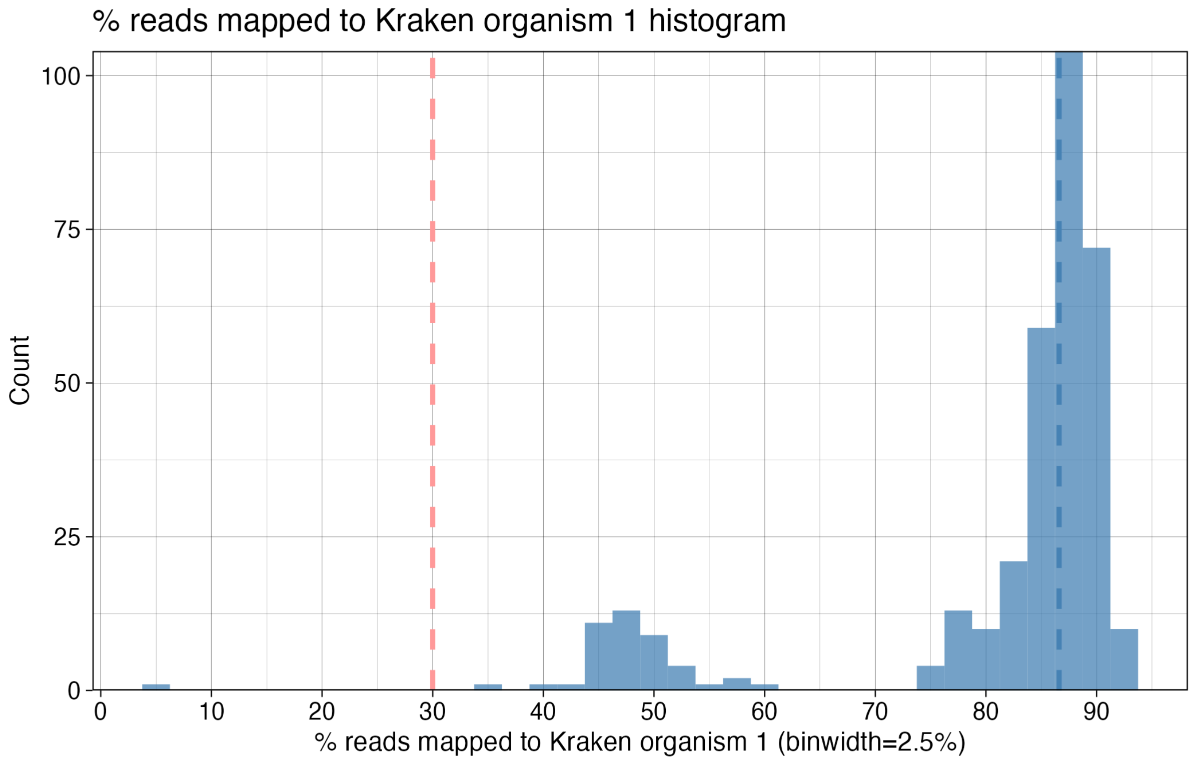

Histogram of % reads mapping to Kraken organism 1 for the 338 samples pre-QC filtering. Dotted vertical lines show median (blue) and QC cut-off (>30%, red).

Supplementary Figure 6

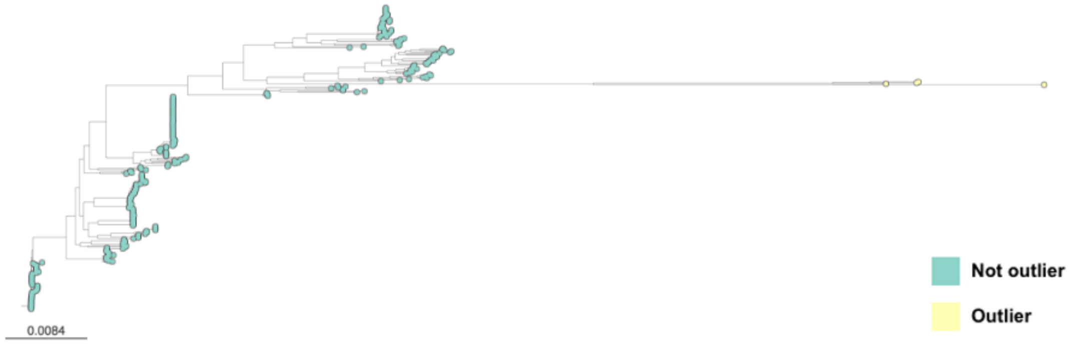

Phylogenetic tree of 326 samples following initial QC filtering. Core genome alignment performed using *roary* (requiring genes to be present in 99% samples), followed by tree generation from the core alignment file using IQTREE as described in Methods. Four samples were phylogenetic outliers - coloured pale yellow on the tree. The four samples had relatively low % reads mapping to kraken 1 (*E. coli*), ranging from 38% (the lowest of the 326 samples included post-QC) to 47%. In all four cases, kraken 2 was *Salmonella enterica*. It is possible these samples are mixed with non-*E. coli* organisms, causing the long branch. The four outliers were removed to yield the final analysis set of 322 samples described in the paper main text. Image downloaded from microreact.

Supplementary Figure 7

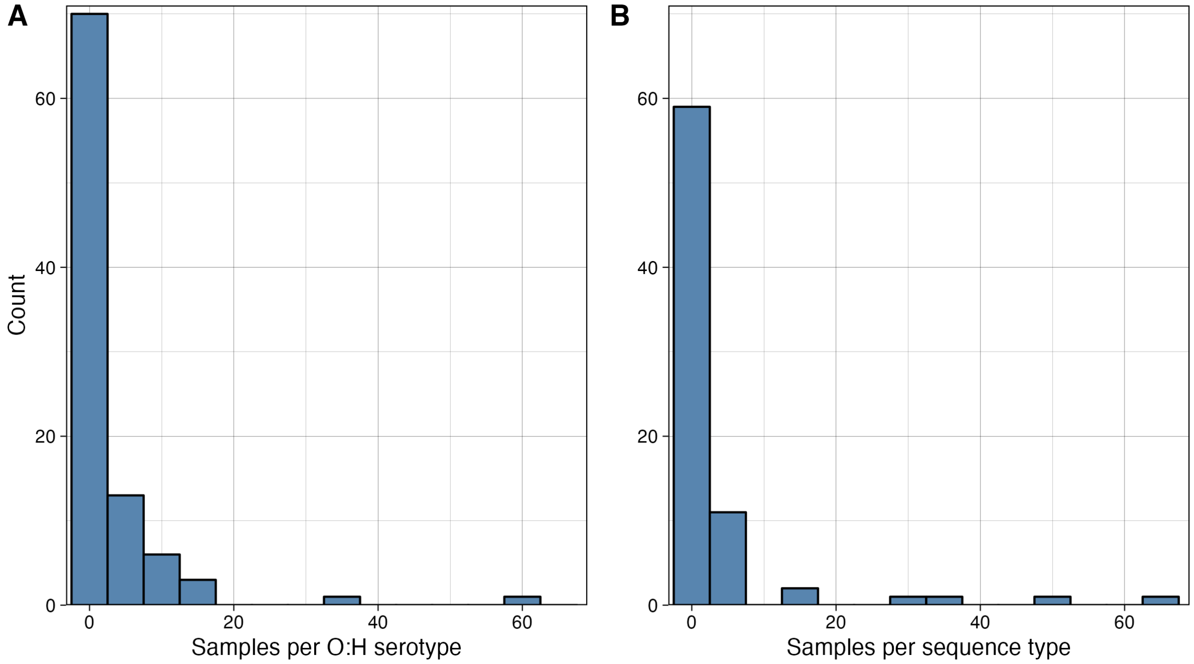

Histograms of O:H serotype (A) and sequence type (B) frequencies for the 322 *E. coli* genomes in the study. Histogram bin width = 5 for both plots. A small minority of both O:H serotypes and sequence types include many samples – the most frequent O:H serotype, O25:H4, includes 60 (18.6%) samples, while 58 O:H serotypes are only represented by a single sample. Similarly, the most frequent sequence type, ST131, includes 65 (20.2%) samples, while 42 sequence types are only represented by a single sample.

Supplementary Figure 8

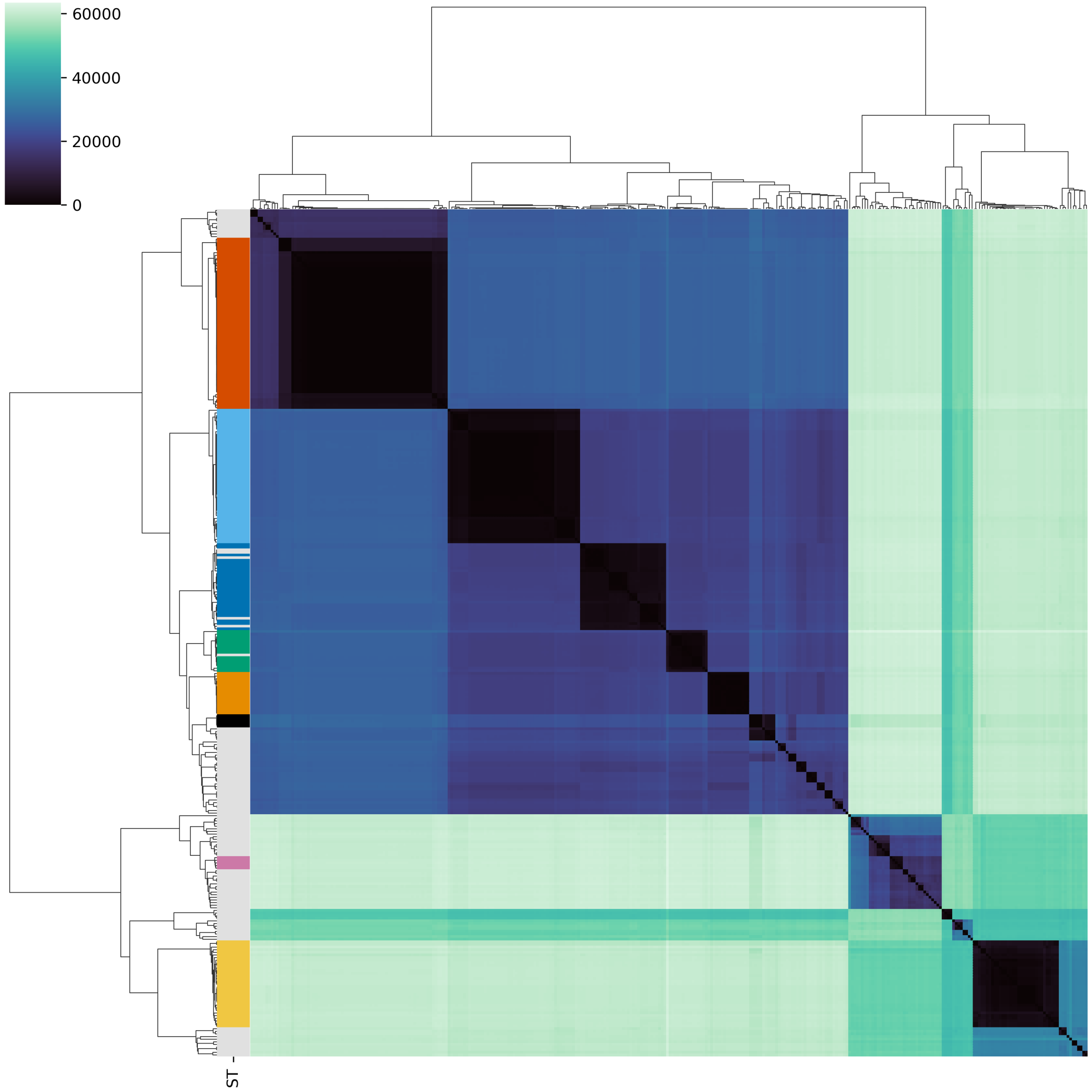

SNP difference distance matrix highlighting major sequence types (coloured bar, right-hand side). There were low pairwise SNP difference counts between samples within each sequence type. SNP differences were counted from the core genome alignment (produced using *roary*), using *snp-dists*.

Supplementary Figure 9

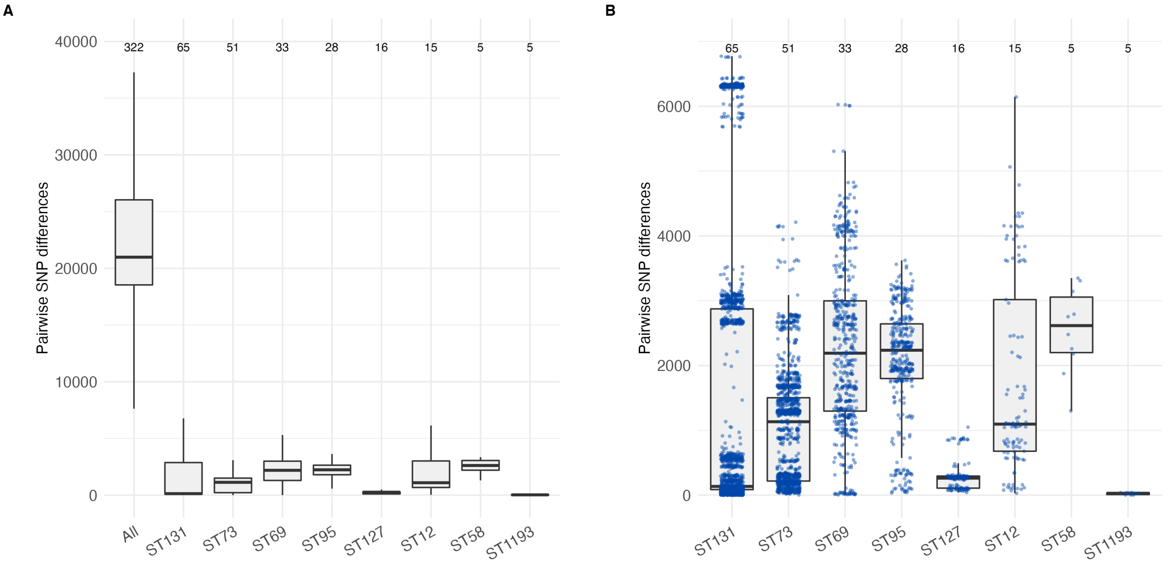

Pairwise SNP differences between isolates for different samples grouped by Sequence Type (ST).

Supplementary Figure 10

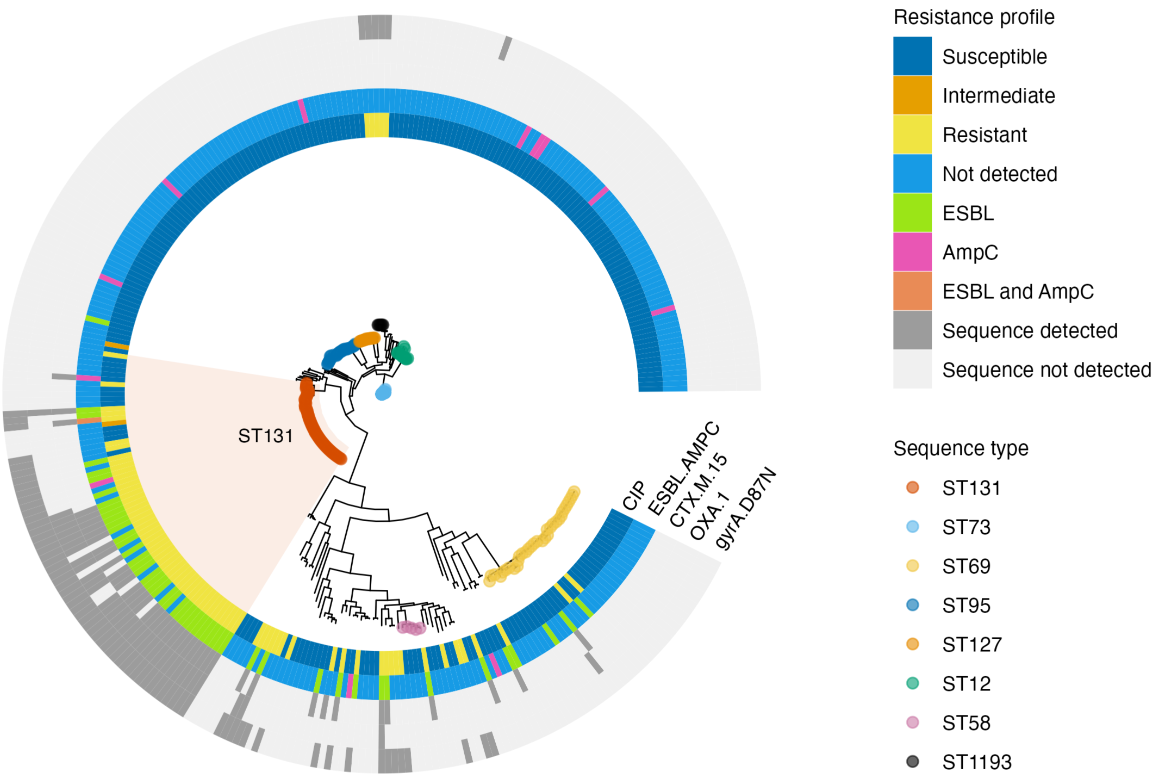

Core genome phylogeny highlighting sequence type 131, which was associated with ESBL production and ciprofloxacin resistance phenotypically, and high rates of *bla*-CTX-M-15, *bla*-OXA-1, and *gyrA*-D87N mutation detection genetically. CIP = ciprofloxacin.

Supplementary Figure 11

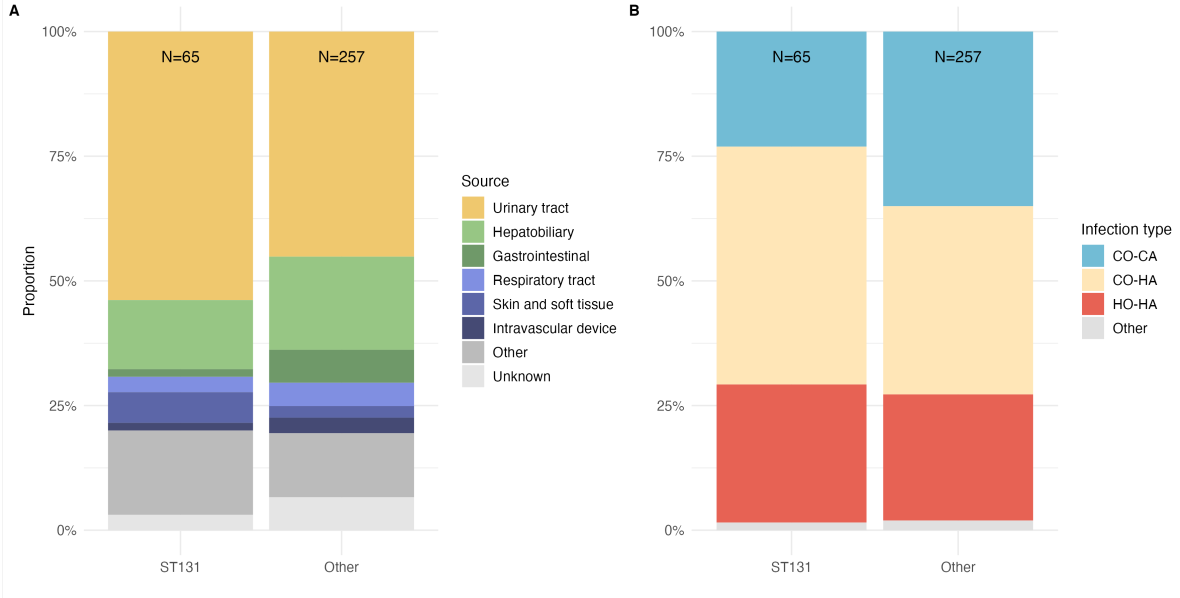

Bacteraemia source and infection class for ST131 vs non-ST131 isolates. CO-CA = Community-onset, Community Associated; CO-HA = Community-onset, Healthcare-associated; HO-HA = Hospital-onset, Healthcare-associated.

Supplementary tables

Supplementary Table 1

	Description	Counts
**Core genes**	99% <= strains <= 100%	2656
**Soft core genes**	95% <= strains < 99%	316
**Shell genes**	15% <= strains < 95%	3377
**Cloud genes**	0% <= strains < 15%	24588
**Total genes**	0% <= strains <= 100%	30937

Output from *roary* for core genome alignment of 322 samples included in the main analysis. *Roary* paramaters required genes to be present in 99% samples.

Supplementary Table 2

		Died	Survived	Whole cohort
**Number of patients**		38	275	313
**Sex**	Female	24 (63%)	144 (52%)	168
**Age (years)**	Median (IQR)	77 (67 – 88)	74 (59 – 84)	75 (60 – 85)
**Infection type**	CO-CA<br>CO-HA<br>HO-HA	13 (34%)<br>14 (37%)<br>11 (29%)	90 (33%)<br>114 (41%)<br>70 (25%)	103<br>128<br>81
**Source**	UTI<br>Non-UTI	5 (13%)<br>33 (87%)	144 (52%)<br>131 (48%)	149<br>164
**ESBL and/or AmpC**	ESBL/AmpC present	8 (21%)	55 (20%)	63
**Sequence type**	ST131	9 (24%)	55 (20%)	64

Breakdown of adult patients that died or survived at 30-days post *E. coli* bacteraemia. (Total cohort = 322; of which 318 adults). CO-CA = Community onset, community-associated; CO-HA = Community onset, healthcare-associated; HO-HA = Hospital onset, healthcare-associated.

Supplementary Table 3

Sequence type	count	%
131	65	20.2
73	51	15.8
69	33	10.2
95	28	8.7
127	16	4.97
12	15	4.66
1193	5	1.55
58	5	1.55

117	4	1.24
144	4	1.24
10	3	0.932
141	3	0.932
28	3	0.932
372	3	0.932
405	3	0.932
80	3	0.932
88	3	0.932
1231	2	0.621
135	2	0.621
14	2	0.621
1406	2	0.621
162	2	0.621
362	2	0.621
367	2	0.621
38	2	0.621
404	2	0.621
428	2	0.621
453	2	0.621
533	2	0.621
648	2	0.621
681	2	0.621
83	2	0.621
1041	1	0.311
108	1	0.311
1141	1	0.311
1257	1	0.311
1266	1	0.311
1385	1	0.311
1431	1	0.311
1434	1	0.311
1485	1	0.311
1590	1	0.311
1597	1	0.311
1656	1	0.311
1876*	1	0.311
191	1	0.311
2015	1	0.311
2227	1	0.311
224	1	0.311
2554	1	0.311

2599	1	0.311
3177	1	0.311
354	1	0.311
354*	1	0.311
3556	1	0.311
357	1	0.311
359	1	0.311
390	1	0.311
398	1	0.311
4088	1	0.311
410	1	0.311
421	1	0.311
537	1	0.311
538	1	0.311
569	1	0.311
5891	1	0.311
59	1	0.311
607	1	0.311
625	1	0.311
640	1	0.311
652	1	0.311
6999	1	0.311
7092	1	0.311
783	1	0.311
8915	1	0.311
998	1	0.311
NF*	1	0.311

List of sequence types by frequency, as defined using *srst2*. 4 sequence types accounted for 55% of all isolates: ST131, ST73, ST69, and ST95.

Supplementary Table 4

AMR gene					Isolate sequence type			
**CTX-M-1**	**CTX-M-14**	**CTX-M-15**	**CTX-M-27**	**OXA-1**	**ST131 (n=65), counts**	**ST131 (n=65), %**	**Non-ST131 (n=257), counts**	**Non-ST131 (n=257), %**
0	0	1	0	1	30	46.2%	2	0.8%
0	0	1	0	0	4	6.2%	9	3.5%
0	0	0	1	0	6	9.2%	0	0.0%
0	0	0	0	1	4	6.2%	2	0.8%
1	0	0	0	0	0	0.0%	1	0.4%
0	1	0	0	0	0	0.0%	3	1.2%
0	0	0	0	0	21	32.3%	240	93.4%
**Total**					**65**		**257**	

Sample counts for carrying the AMR genes CTX-M and OXA-1, for ST131 (n=65) and non-ST131 (n=257) isolates. 1 = AMR gene detected; 0 = AMR gene not detected.

Supplementary Table 5

Genotype			Ciprofloxacin susceptibility phenotype			
***gyrA*-S83L**	***gyrA*-D87N**	***parC*-S80I**	**Susceptible (n=241), counts**	**Susceptible (n=241), %**	**Resistant (n=79), counts**	**Resistant (n=79), %**
0	0	0	214	88.8%	1	1.3%
0	1	0	1	0.4%	3	3.8%
1	0	0	25	10.4%	4	5.1%
1	1	1	1	0.4%	71	89.9%
**Total**			**241**		**79**	

Sample counts for carrying key *E. coli* fluoroquinolone resistance-associated mutations (*gyrA*-S83L, *gyrA*-D87N, and *parC*-S80I), for phenotypically ciprofloxacin susceptible (n=241) and resistant (n=79) isolates. The 2 isolates reported as phenotypically intermediate are not shown. 1 = variant detected; 0 = variant not detected.

Supplementary Table 6

O:H serotype	count	%
O25:H4	60	18.6
O6:H1	33	10.2
O1:H7	15	4.66
O6:H31	15	4.66
O17:H18	13	4.04
O4:H5	10	3.11
O117:H4	9	2.8
O18:H7	8	2.48
O2:H1	8	2.48
O75:H5	8	2.48
O16:H5	5	1.55
O2:H4	5	1.55
O4:H1	5	1.55
O15:H6	4	1.24
O16:H6	4	1.24
O2:H6	4	1.24
O2:H7	4	1.24
O8:H10	4	1.24
O129:H4	3	0.932
O18:H1	3	0.932
O75:H7	3	0.932
O83:H1	3	0.932
O86:H18	3	0.932
O1:H6	2	0.621
O102:H6	2	0.621
O153:H2	2	0.621
O153:H4	2	0.621
O161:H4	2	0.621
O18:H31	2	0.621
O18:H5	2	0.621
O22:H1	2	0.621
O23:H16	2	0.621
O51:H14	2	0.621
O6:H5	2	0.621
O7:H6	2	0.621
O10:H12	1	0.311
O102:H23	1	0.311
O104:H9	1	0.311
O108:H2	1	0.311
O109:H10	1	0.311

O11:H4	1	0.311
O112:H19	1	0.311
O113:H4	1	0.311
O12:H4	1	0.311
O120:H15	1	0.311
O120:H31	1	0.311
O129:H15	1	0.311
O131:H12	1	0.311
O134:H31	1	0.311
O138:H34	1	0.311
O143:H4	1	0.311
O15:H1	1	0.311
O15:H18	1	0.311
O15:H4	1	0.311
O153:H15	1	0.311
O153:H25	1	0.311
O153:H34	1	0.311
O153:H6	1	0.311
O157:H39	1	0.311
O160:H21	1	0.311
O160:H40	1	0.311
O168:H25	1	0.311
O170:H7	1	0.311
O18:H14	1	0.311
O2:H14	1	0.311
O2:H31	1	0.311
O21:H5	1	0.311
O25:H1	1	0.311
O25:H18	1	0.311
O33:H6	1	0.311
O45:H7	1	0.311
O48:H20	1	0.311
O5:H18	1	0.311
O6:H7	1	0.311
O64:H20	1	0.311
O7:H15	1	0.311
O7:H45	1	0.311
O76:H14	1	0.311
O76:H6	1	0.311
O8:H19	1	0.311
O8:H20	1	0.311
O8:H25	1	0.311

O8:H4	1	0.311
O8:H8	1	0.311
O83:H42	1	0.311
O83:H5	1	0.311
O85:H18	1	0.311
O9:H19	1	0.311
O9:H25	1	0.311
O9:H4	1	0.311
O9:H5	1	0.311
O9:H9	1	0.311
O96:H23	1	0.311
Missing or incomplete serotype call	11	3.42

List of O:H serotypes by frequency, as defined using *srst2*.

Supplementary Table 7

ENA accession code	Sequence type	OH prediction	Anti-O25b antibody binding
ERS4216483	10	O15:H4	
ERS4216606	10	O12:H4	
ERS4216298	10	O160:H21	
ERS4216421	12	O4:H5	
ERS4216562	12	O4:H5	
ERS4216453	12	O4:H1	
ERS4216282	12	O4:H1	
ERS4216525	12	O4:H5	
ERS4216368	12	O21:H5	
ERS4216507	12	O4:H5	
ERS4216227	12	O4:H5	
ERS4216472	12	O4:H1	
ERS4216299	12	O4:H1	
ERS4216497	12	O4:H1	
ERS4216244	12	O4:H5	
ERS4216409	12	O4:H5	
ERS4216248	12	O4:H5	
ERS4216257	12	O4:H5	
ERS4216486	14	O18:H5	
ERS4216435	14	O18:H5	
ERS4216228	28	Missing or incomplete OH call	
ERS4216376	28	O33:H6	
ERS4216312	28	O76:H6	
ERS4216433	38	O86:H18	
ERS4216477	38	O7:H15	
ERS4216225	58	O9:H9	
ERS4216307	58	O8:H25	
ERS4216331	58	O168:H25	
ERS4216356	58	O153:H25	
ERS4216406	58	O9:H25	
ERS4216631	59	O1:H7	
ERS4216395	69	O117:H4	
ERS4216420	69	O15:H1	
ERS4216434	69	O117:H4	
ERS4216445	69	O17:H18	
ERS4216575	69	O17:H18	
ERS4216459	69	O17:H18	

ERS4216469	69	O17:H18	
ERS4216287	69	O5:H18	
ERS4216222	69	O15:H18	
ERS4216333	69	O153:H2	
ERS4216540	69	O17:H18	
ERS4216369	69	O117:H4	
ERS4216218	69	O117:H4	
ERS4216633	69	O15:H6	
ERS4216290	69	O17:H18	
ERS4216297	69	O86:H18	
ERS4216236	69	O17:H18	
ERS4216548	69	O17:H18	
ERS4216587	69	O153:H2	
ERS4216313	69	O86:H18	
ERS4216317	69	O11:H4	
ERS4216322	69	O117:H4	
ERS4216693	69	O117:H4	
ERS4216240	69	O117:H4	
ERS4216350	69	O17:H18	
ERS4216386	69	O15:H6	
ERS4216397	69	O17:H18	
ERS4216424	69	O17:H18	
ERS4216426	69	O25:H18	
ERS4216252	69	O17:H18	
ERS4216425	69	O17:H18	
ERS4216232	69	O15:H6	
ERS4216460	69	O15:H6	
ERS3033947	73	O6:H1	
ERS4216220	73	O6:H1	
ERS4216399	73	O18:H1	
ERS4216422	73	O18:H1	
ERS4216429	73	O6:H1	
ERS4216561	73	O6:H1	
ERS4216438	73	O6:H1	
ERS4216563	73	O18:H1	
ERS4216571	73	O120:H31	
ERS4216580	73	O6:H1	
ERS4216464	73	O6:H1	
ERS4216274	73	O6:H1	
ERS4216461	73	O6:H1	
ERS4216467	73	O6:H1	
ERS4216590	73	O22:H1	

ERS4216495	73	O6:H1	
ERS4216491	73	Missing or incomplete OH call	
ERS4216332	73	O6:H1	
ERS4216355	73	O6:H1	
ERS4216223	73	O6:H1	
ERS4216535	73	O6:H1	
ERS4216536	73	O2:H1	
ERS4216363	73	O6:H1	
ERS4216498	73	O2:H1	
ERS4216504	73	O2:H1	
ERS4216375	73	O6:H1	
ERS4216506	73	O6:H1	
ERS4216509	73	O2:H1	
ERS4216390	73	O22:H1	
ERS4216226	73	O6:H1	
ERS4216692	73	Missing or incomplete OH call	
ERS4216221	73	O6:H1	
ERS4216608	73	O6:H1	
ERS4216612	73	O2:H1	
ERS4216593	73	O2:H1	
ERS4216475	73	O6:H1	
ERS4216517	73	O6:H1	
ERS4216304	73	O6:H1	
ERS4216523	73	O6:H1	
ERS4216576	73	O6:H1	
ERS4216597	73	O6:H1	
ERS4216616	73	O6:H1	
ERS4216617	73	O2:H1	
ERS4216620	73	O25:H1	
ERS4216326	73	O6:H1	
ERS4216328	73	O2:H1	
ERS4216230	73	O6:H1	
ERS4216408	73	O6:H1	
ERS4216416	73	Missing or incomplete OH call	
ERS4216455	73	O6:H1	
ERS4216288	73	O6:H1	
ERS4216412	80	O75:H7	
ERS4216586	80	O75:H7	

ERS4216512	80	O75:H7	
ERS4216224	83	O83:H5	
ERS4216614	83	O6:H5	
ERS4216503	88	O8:H4	
ERS4216489	88	O9:H4	
ERS4216443	88	O160:H40	
ERS4216515	95	O2:H7	
ERS4216541	95	O1:H7	
ERS4216423	95	O2:H4	
ERS4216432	95	O18:H7	
ERS4216558	95	O25:H4	
ERS4216437	95	O1:H7	
ERS4216441	95	O1:H7	
ERS4216569	95	O18:H7	
ERS4216573	95	O18:H7	
ERS4216583	95	O18:H7	
ERS4216285	95	O2:H4	
ERS4216499	95	O1:H7	
ERS4216505	95	O1:H7	
ERS4216519	95	O25:H4	
ERS4216588	95	O1:H7	
ERS4216592	95	O2:H4	
ERS4216626	95	O2:H7	
ERS4216628	95	O1:H7	
ERS4216478	95	O1:H7	
ERS4216502	95	O18:H7	
ERS4216327	95	O1:H7	
ERS4216346	95	O1:H7	
ERS4216262	95	O2:H4	
ERS4216389	95	O1:H7	
ERS4216273	95	O2:H7	
ERS4216249	95	O45:H7	
ERS4216254	95	O2:H7	
ERS4216468	95	O1:H7	
ERS4216479	108	O129:H15	
ERS4216308	117	O161:H4	
ERS4216336	117	O161:H4	
ERS4216604	117	O143:H4	
ERS4216318	117	O85:H18	
ERS3033948	127	O6:H31	
ERS4216551	127	O6:H31	
ERS4216579	127	O6:H31	

ERS4216253	127	Missing or incomplete OH call	
ERS4216493	127	O6:H31	
ERS4216344	127	O6:H31	
ERS4216359	127	O6:H31	
ERS4216632	127	O6:H31	
ERS4216643	127	O6:H31	
ERS4216306	127	O6:H31	
ERS4216238	127	O6:H31	
ERS4216381	127	O6:H31	
ERS4216264	127	O6:H31	
ERS4216391	127	O6:H31	
ERS4216361	127	O6:H31	
ERS4216263	127	O6:H31	
ERS3033949	131	O25:H4	Strong binding
ERS3033950	131	O25:H4	Strong binding
ERS4216242	131	O25:H4	Strong binding
ERS4216398	131	O25:H4	Strong binding
ERS4216410	131	O25:H4	Strong binding
ERS4216544	131	O25:H4	Strong binding
ERS4216418	131	O25:H4	Strong binding
ERS4216547	131	O16:H5	No binding
ERS4216566	131	O25:H4	Strong binding
ERS4216447	131	O25:H4	Strong binding
ERS4216448	131	O25:H4	No binding
ERS4216458	131	O25:H4	Strong binding
ERS4216457	131	O25:H4	Strong binding
ERS4216582	131	O25:H4	Strong binding
ERS4216584	131	O25:H4	Strong binding
ERS4216494	131	O16:H5	No binding
ERS4216501	131	O25:H4	Strong binding
ERS4216520	131	O25:H4	Strong binding
ERS4216526	131	O25:H4	Strong binding
ERS4216340	131	O25:H4	Strong binding
ERS4216343	131	O25:H4	Strong binding
ERS4216534	131	O25:H4	No binding
ERS4216537	131	O25:H4	No binding
ERS4216357	131	O25:H4	No binding
ERS4216538	131	O25:H4	Strong binding
ERS4216372	131	O16:H5	No binding
ERS4216595	131	O25:H4	Strong binding
ERS4216596	131	O25:H4	Strong binding

ERS4216217	131	O25:H4	Strong binding
ERS4216611	131	O25:H4	Strong binding
ERS4216622	131	O25:H4	Strong binding
ERS4216594	131	O25:H4	Strong binding
ERS4216629	131	O25:H4	No binding
ERS4216589	131	O25:H4	Strong binding
ERS4216638	131	O25:H4	Strong binding
ERS4216640	131	O25:H4	Strong binding
ERS4216639	131	O25:H4	Strong binding
ERS4216295	131	O25:H4	Strong binding
ERS4216365	131	O25:H4	Strong binding
ERS4216234	131	O25:H4	Strong binding
ERS4216303	131	O25:H4	Strong binding
ERS4216521	131	O25:H4	Strong binding
ERS4216305	131	O25:H4	Strong binding
ERS4216524	131	O25:H4	No binding
ERS4216367	131	O25:H4	Strong binding
ERS4216316	131	O25:H4	Strong binding
ERS4216325	131	O25:H4	Strong binding
ERS4216324	131	O25:H4	Strong binding
ERS4216239	131	O25:H4	Strong binding
ERS4216329	131	O25:H4	Strong binding
ERS4216330	131	O25:H4	Strong binding
ERS4216335	131	O25:H4	Strong binding
ERS4216334	131	O25:H4	Strong binding
ERS4216338	131	O16:H5	No binding
ERS4216342	131	O25:H4	Strong binding
ERS4216345	131	O25:H4	No binding
ERS4216348	131	O16:H5	No binding
ERS4216349	131	O25:H4	Strong binding
ERS4216379	131	O153:H4	No binding
ERS4216380	131	O25:H4	Strong binding
ERS4216266	131	O25:H4	Strong binding
ERS4216269	131	O153:H4	No binding
ERS4216281	131	O25:H4	Strong binding
ERS4216430	131	O25:H4	Strong binding
ERS4216289	131	O25:H4	Strong binding
ERS4216557	135	O83:H1	
ERS4216598	135	O83:H1	
ERS4216601	141	O2:H6	
ERS4216277	141	O2:H6	
ERS4216235	141	O2:H6	

ERS4216272	144	O16:H6	
ERS4216518	144	O16:H6	
ERS4216267	144	O16:H6	
ERS4216411	144	O16:H6	
ERS4216388	162	O109:H10	
ERS4216402	162	O8:H10	
ERS4216466	191	O48:H20	
ERS4216293	224	O102:H23	
ERS4216546	354	O153:H34	
ERS4216354	354	O153:H15	
ERS4216444	357	O129:H4	
ERS4216619	359	O131:H12	
ERS4216431	362	O7:H6	
ERS4216250	362	O7:H6	
ERS4216405	367	Missing or incomplete OH call	
ERS4216449	367	O9:H19	
ERS4216450	372	O18:H31	
ERS4216644	372	O2:H31	
ERS4216294	372	O18:H31	
ERS4216621	390	O18:H7	
ERS4216300	398	O8:H20	
ERS4216568	404	O75:H5	
ERS4216260	404	O75:H5	
ERS4216446	405	O2:H4	
ERS4216531	405	O102:H6	
ERS4216302	405	O102:H6	
ERS4216374	410	Missing or incomplete OH call	
ERS4216404	421	O1:H7	
ERS4216462	428	O117:H4	
ERS4216373	428	O117:H4	
ERS4216451	453	O23:H16	
ERS4216609	453	O23:H16	
ERS4216496	533	O64:H20	
ERS4216229	533	O76:H14	
ERS4216641	537	O75:H5	
ERS4216258	538	O129:H4	
ERS4216484	569	O134:H31	
ERS4216625	607	O10:H12	
ERS4216296	625	O6:H7	

ERS4216488	640	O129:H4	
ERS4216578	648	O1:H6	
ERS4216510	648	O153:H6	
ERS4216615	652	Missing or incomplete OH call	
ERS4216542	681	O8:H10	
ERS4216508	681	O8:H10	
ERS4216320	783	O104:H9	
ERS4216528	998	O2:H6	
ERS4216360	1041	O157:H39	
ERS4216550	1141	O113:H4	
ERS4216442	1193	O75:H5	
ERS4216476	1193	O75:H5	
ERS4216255	1193	O75:H5	
ERS4216695	1193	O75:H5	
ERS4216624	1193	O75:H5	
ERS4216599	1231	O18:H7	
ERS4216366	1231	O18:H7	
ERS4216618	1257	O8:H10	
ERS4216339	1266	O138:H34	
ERS4216529	1385	Missing or incomplete OH call	
ERS4216481	1406	O51:H14	
ERS4216645	1406	O51:H14	
ERS4216436	1431	O8:H19	
ERS4216414	1434	O18:H14	
ERS4216630	1485	O83:H42	
ERS4216321	1590	O120:H15	
ERS4216394	1597	Missing or incomplete OH call	
ERS4216607	1656	O8:H8	
ERS4216301	1876	Missing or incomplete OH call	
ERS4216603	2015	O2:H14	
ERS4216275	2227	O9:H5	
ERS4216337	2554	O83:H1	
ERS4216276	2599	O108:H2	
ERS4216407	3177	O1:H6	
ERS4216623	3556	O96:H23	
ERS4216261	4088	O170:H7	
ERS4216341	5891	O112:H19	

ERS4216559	6999	O1:H7	
ERS4216268	7092	O6:H5	
ERS4216585	8915	O7:H45	
ERS4216602	NA	O4:H5	

ENA accession codes for the 322 isolates analysed in this study. Also showing the predicted sequence type and OH serogroup (by *srst2*) and antibody binding profile, tested on the 65 ST131 isolates.
